# Fine mapping of the *BnUC2* locus related to leaf up-curling and plant semi-dwarfing in *Brassica napus*

**DOI:** 10.1186/s12864-020-06947-7

**Published:** 2020-07-31

**Authors:** Chengwei Huang, Mao Yang, Danlei Shao, Yangming Wang, Shubei Wan, Jianbo He, Zuqing Meng, Rongzhan Guan

**Affiliations:** 1 National Key Laboratory of Crop Genetics and Germplasm Enhancement, Jiangsu Collaborative Innovation Center for Modern Crop Production, Nanjing Agricultural University, Nanjing, 210095 China; 2 Tibet Agriculture and Animal Husbandry College, Linzhi, 860000 Tibet Autonomous Region China

**Keywords:** *Brassica napus*, Up-curled leaves, Semi-dwarf, Single nucleotide polymorphism, Simple sequence repeat, Gene mapping

## Abstract

**Background:**

Studies of leaf shape development and plant stature have made important contributions to the fields of plant breeding and developmental biology. The optimization of leaf morphology and plant height to improve lodging resistance and photosynthetic efficiency, increase planting density and yield, and facilitate mechanized harvesting is a desirable goal in *Brassica napus*.

**Results:**

Here, we investigated a *B. napus* germplasm resource exhibiting up-curled leaves and a semi-dwarf stature. In progeny populations derived from NJAU5737 and Zhongshuang 11 (ZS11), we found that the up-curled leaf trait was controlled by a dominant locus, *BnUC2*. We then fine mapped the *BnUC2* locus onto an 83.19-kb interval on chromosome A05 using single nucleotide polymorphism (SNP) and simple sequence repeat (SSR) markers. We further determined that *BnUC2* was a major plant height QTL that explained approximately 70% of the phenotypic variation in two BC_5_F_3_ family populations derived from NJAU5737 and ZS11. This result implies that *BnUC2* was also responsible for the observed semi-dwarf stature*.* The fine mapping interval of *BnUC2* contained five genes, two of which, *BnaA05g16700D* (*BnaA05.IAA2*) and *BnaA05g16720D,* were revealed by comparative sequencing to be mutated in NJAU5737. This result suggests that the candidate gene mutation (*BnaA05g16700D*, encoding Aux/IAA2 proteins) in the conserved Degron motif GWPPV (P63S) was responsible for the *BnUC2* locus. In addition, investigation of agronomic traits in a segregated population indicated that plant height, main inflorescence length, and branching height were significantly reduced by *BnUC2*, whereas yield was not significantly altered. The determination of the photosynthetic efficiency showed that the *BnUC2* locus was beneficial to improve the photosynthetic efficiency. Our findings may provide an effective foundation for plant type breeding in *B. napus*.

**Conclusions:**

Using SNP and SSR markers, a dominant locus (*BnUC2*) related to up-curled leaves and semi-dwarf stature in *B. napus* has been fine mapped onto an 83.19-kb interval of chromosome A05 containing five genes. The *BnaA05.IAA2* is inferred to be the candidate gene responsible for the *BnUC2* locus.

## Background

Leaf morphology and plant stature are determining factors for dicot plant photosynthesis, dry mater accumulation, lodging resistance, tolerance to high planting density, and amenability to mechanized harvesting. Slight up-curling of leaves and a semi-dwarf stature may improve grain yield [1, 2]. Research on leaf morphology and plant stature is important in the fields of plant developmental biology and crop genetic improvement. Leaf development from the shoot apical meristem comprises several stages, including leaf primordium formation, polarity establishment, and cell differentiation. Leaf curling is due to abnormal leaf development caused by mutations of genes related to leaf development [3–5]. Many genes have been identified to be involved in leaf development. Transcription factors, including KANADI [6–9], Class III HOMEODOMAIN LEUCINE-ZIPPER (HD-Zip III) [10–13], WUSCHEL RELATED HOMEOBOX (WOX) [14], and TB1-CYC-PCFs (TCPs) [15], participate in alteration of leaf polarity establishment, which leads to the up-curled leaf phenotype. Plant hormone biosynthesis and signal transduction is another major aspect that influences leaf shape. AUXIN/INDOLE-3-ACETIC ACID (Aux/IAA) gene family mutations have been reported to cause leaf curling in plants [16–19]. The activity of auxin response factors (ARFs) affects the mutual antagonism between HD-ZIP IIIs and KANADI that regulates leaf development. Mutation of *Arabidopsis ARF3* can cause leaf curling [20]. The *UCU1* gene encoding the SHAGGY/GSK3 protein involved in the signal transduction of auxin and BR can lead to down-curled leaves and short stature phenotypes [21]. In addition, some microRNAs that can specifically recognize the START domain of HD-ZIP III family genes, such as miRNA165 and miRNA166, may cause leaf curling by regulating HD-ZIP III family gene expression [11, 22–24]. miRNA160 regulates leaf curling by controlling the expressions of auxin-responsive genes ARF10 and ARF17 [25, 26]. miRNA164 affects the development of leaf margins by regulating the expression of the *CUC1* gene, which may lead to a curled leaf phenotype [27].

Plant height is mainly determined by plant hormone biosynthesis, signal transduction, and related pathways. Although not described here, a well-documented relationship exists between the regulation of plant dwarf stature and genes involved in gibberellin and brassinosteroid biosynthesis and signal transduction pathways [28–33]. Auxin biosynthesis, polar transport, and signal transduction exert a crucial role in plant growth and development, including plant height determination. *YUCCA* encodes a flavin monooxygenase, a catalytic enzyme in the auxin biosynthesis pathway, and mutation of *YUCCA* leads to short stature in plants [34, 35]. Phosphorylated glycoproteins PGP1 and PGP19/MDR1 are vectors for auxin transport. Because of their weaker auxin polar transport capacities, the *mdr1–1* single mutant and *mdr1–1/pgp1–1* double mutant of *Arabidopsis* are dwarfs [36, 37]. The auxin signal transduction pathway is mainly composed of transport inhibitor resistant1/auxin signaling F-box proteins (TIR1/AFBs), Aux/IAA proteins, and ARFs [38–40]. Aux/IAA proteins, which act as repressors of auxin-regulated transcriptional activation, possess four conserved domains (domains I, II, III, and IV), and domain II contains a strongly conserved amino acid motif, GWPPV. In the presence of auxin, TIR1/AFBs can recognize this motif and bind to the Aux/IAA proteins, leading to the degradation of Aux/IAA proteins. ARF is then released to activate the expressions of auxin-response genes [41, 42]. The GWPPV motif, called the Degron domain, is thus the core component of auxin signal transduction [43–46]. Mutations of any sequence in the GWPPV motif or its flanks can lead to defective plant growth and development, including the generation of shorter hypocotyls and stature, leaf curling, reduction in the number of lateral roots, and loss of apical dominance [47–49]. In *Arabidopsis* and *B. napus*, mutations of Aux/IAA genes decrease plant height and lead to leaf curling [17, 50–52].

Curled-leaf traits, including up-curling, down-curling, and wrinkling, are often observed in *B. napus*. Leaf up-curling is a useful trait that allows an increase in the planting density of *B. napus.* Published research on curled-leaf traits is limited. Wang et al. [53] found that the wrinkled, down-curved leaf type of *Bndwf/dcll*, which is short-statured, is controlled by a dominant gene. Li et al. [51] reported a *B. napus* mutant, *sca*, with crinkled leaves, a semi-dwarf stature, narrow branch angles, and upright siliques, and determined that the underlying gene related to the mutated traits is a semi-dominant gene. Zhao et al. [17] discovered that a semi-dominant gene is responsible for an extremely dwarf mutant, *ds-4*, with down-curved leaves. Yang et al. [54] uncovered an up-curling leaf locus (*BnUC1*) associated with a dominant gene.

Because of the important role of dwarf/semi-dwarf plant resources in crop genetic improvement, especially in dicots, their excavation and utilization has attracted the attention of agronomists and biologists. In *B. napus*, the dwarf phenotypes of *bzh* [55] and *NDF-1* [56] mutants are controlled by a major gene with additive effects, while the dwarf trait of 99CDAM [57] is caused by three pairs of recessive genes. Although some mutants related to plant height in *B. napus* have been reported, few genes have been studied to clarify their mechanical roles in plant development. The semi-dwarf nature of gibberellin-insensitive mutants, including *NDF-1* [56], *ds-1* [58], *ds-3* [59] and *banC.dwf* [60], and *ds-1* and *ds-3* are caused by a mutation in the VHYNP motif of the DELLA protein. In *ds-4* [17] and *sca* [51] mutants insensitive to auxin, a mutation in the GWPPV motif of the Aux/IAA7 protein is responsible for their semi-dwarf/dwarf phenotypes. The dwarf characteristics of *banC.dwf* [60], *Bndwf1* [61] and *Bndwf/dcl1* [53] are controlled by a pair of dominant genes, while those of *ds-1* [58], *ds-3* [59], *ds-4* [17] and *sca* [51] are regulated by a single semi-dominant ones. Many dwarfing genes have a negative effect on crop agronomic traits, which restricts the breeding development of dwarf or semi-dwarf varieties [62]. The investigation of dwarf mutants, the identification of new dwarfing genes, and the elucidation of dwarfing mechanisms are therefore crucial for genetic improvement of *B. napus*.

In the present study, we investigated the pure *B. napus* line NJAU5737 (named *Bnuc2)*, a new semi-dwarf mutant with up-curled leaves developed in our laboratory, analyzed the inheritance of the up-curled leaf trait, and fine mapped the *BnUC2* locus. We also evaluated the effects of the *BnUC2* locus on agronomic traits. Our results may provide an effective foundation for plant type breeding in *B. napus*. Our findings may also serve as a foundation for the semi-dwarf variety breeding of *B. napus* and exploration of the dwarfing mechanism.

## Results

### Performance of the up-curled-leaf mutant

**Fig. 1:**
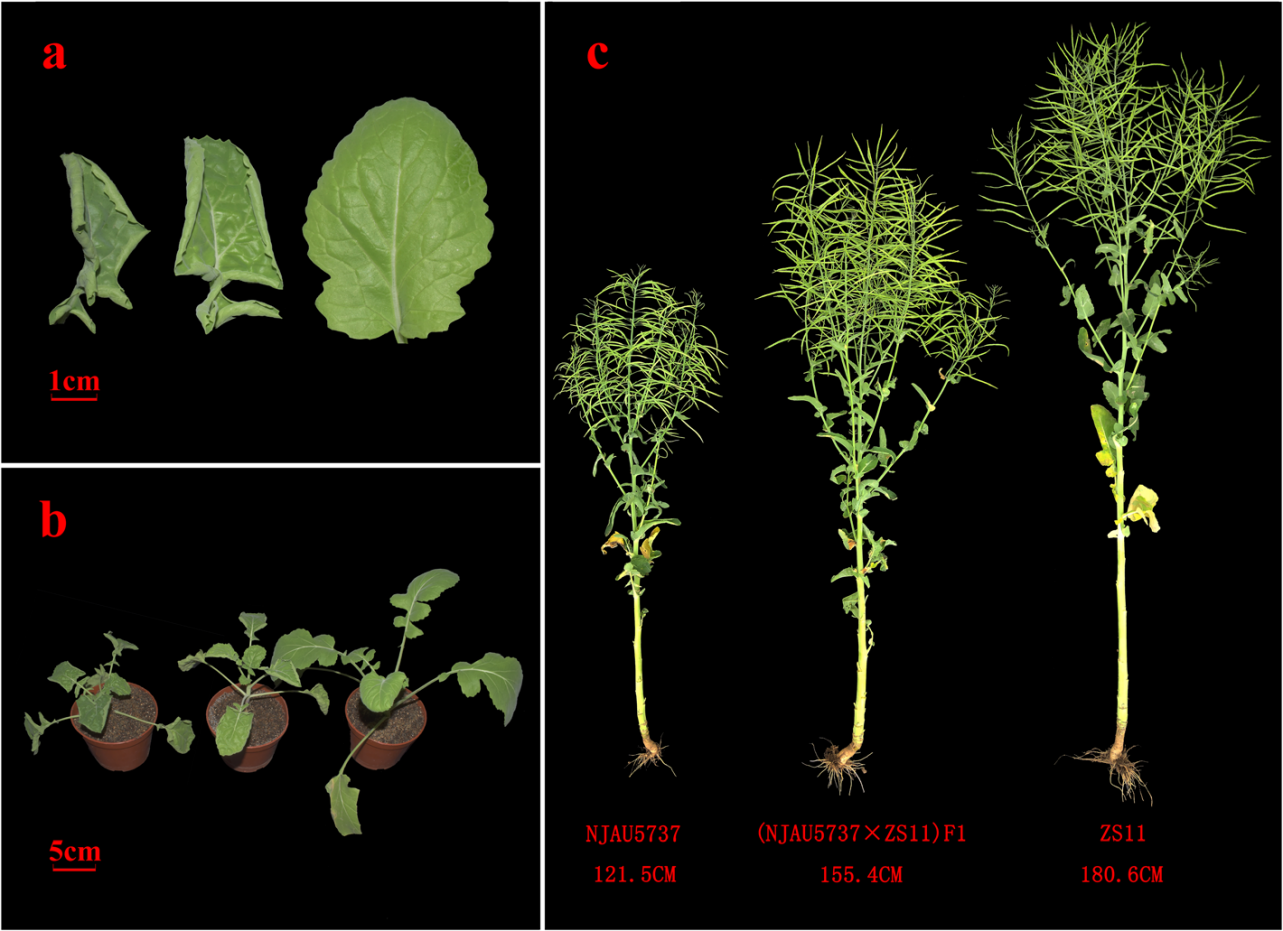
Performance of NJAU5737, (NJAU5737 × ZS11) F_1_ and ZS11. **a** Leaf of NJAU5737 (left), (NJAU5737 × ZS11) F_1_ (middle) and ZS11 (right) at the seedling stage. **b** Seedling of NJAU5737 (left), (NJAU5737 × ZS11) F_1_ (middle) and ZS11 (right). **c** NJAU5737 (left), (NJAU5737 × ZS11) F_1_ (middle) and ZS11 (right) at mature stage

Compared with the Zhongshuang11 (ZS11), the NJAU5737 had shorter hypocotyls and stature. In addition, leaves of NJAU5737 were up-curled and slightly crinkled, whereas ZS11 had normal, flat leaves (Fig. 1a, b). At the mature stage, NJAU5737 plants were approximately 120–130 cm high. F_1_ seedlings of NJAU5737 × ZS11 had up-curled, crinkled leaves (Fig. 1a, b), with mature plant heights that were intermediate between those of the two parents (Fig. 1c).

The leaf Chl a, Chl b, and total Chl contents and Chl a/b ratio in up-curled leaves were significantly higher than flat leaves at the seedling stage (Table 1). This result indicated that the up-curled leaf trait was associated with elevating the leaf Chl content, and result in deep-green leaves.

**Table 1:** Leaf chlorophyll contents of up-curled leaf and flat leaf in BC_5_F_3_ population

Phenotype	Chl a content (mg/g)	Chl b content (mg/g)	Total Chl content (mg/g)	Chl a/b ratio
Up-curled leaf	1.37 ± 0.15**	0.47 ± 0.01**	1.83 ± 0.15**	2.94 ± 0.36**
Flat leaf	1.15 ± 0.12	0.43 ± 0.02	1.58 ± 0.12	2.69 ± 0.27

**indicates significant at the 0.01 probability level. Mean ± standard deviation (*SD*) under sample size

The leaf net photosynthetic rate, stomatal conductance and concentration of intercellular CO_2_ of up-curled leaves were significantly higher than those of flat leaves at the seedling stage, and there was no significant difference in the leaf transpiration rate between the two leaf morphology pattern (Table 2). These results imply that the up-curled leaf trait is associated with elevating the photosynthetic efficiency.

**Table 2:** Leaf photosynthetic indicators of up-curled leaf and flat leaf in BC_5_F_3_ population

Phenotype	NPR *μ*mol CO_2_ m^− 2^ s^− 1^	SC mol H_2_O m^− 2^ s^− 1^	ICC μmol CO_2_ mol^− 1^	TR *m*mol H_2_O m^−2^ s^− 1^
Up-curled leaf	12.18 ± 0.78**	0.34 ± 0.02**	398.50 ± 6.37**	2.89 ± 0.37
Flat leaf	9.67 ± 0.51	0.22 ± 0.03	368.36 ± 4.89	2.30 ± 0.36

Data are presented as means ± *SD, n = 6*. ** indicates significant at 0.01 probability level. NPR, SC, ICC and TR denote net photosynthetic rate, stomatal conductance, intercellular CO_2_ concentration and transpiration rate, respectively

### Inheritance of the up-curled leaf trait

Plants in F_1_ (ZS11 × NJAU5737) and RF_1_ (NJAU5737 × ZS11) generations, obtained by crossing NJAU5737 (up-curled leaves) with ZS11 (normal, flat leaves), all possessed up-curled leaves, which implies that the leaf-curling trait is controlled by dominant genes. The segregation ratio of up-curled to normal leaves in all obtained backcross populations with a dominant locus, from BC_1_ to BC_6_ to ZS11, was in good agreement with the expected Mendelian segregation ratio of 1:1 according to Chi-square tests (Table 3). Furthermore, segregation in subsequently selfed BC_5_F_2_ family populations and two BC_5_F_3_ populations was in accord with the expected Mendelian segregation ratio of 3:1 (up-curled vs. normal leaves) (Table 3). These results indicate that leaf up-curling is controlled by a dominant locus (*BnUC2*).

**Table 3:** Genetic segregation analysis of *BnUC2* in populations derived from NJAU5737 and the recurrent parent ZS11 in *Brassica napus*

Population	Up-curled leaf	Flat leaf	Total	Expected ratio	*χ*^2^ value
F_1_	120	0	120		
RF_1_	115	0	115		
BC_1_	60	55	115	1:1	0.22
BC_2_	42	56	98	1:1	2.00
BC_3_	45	57	102	1:1	1.41
BC_4_	58	70	128	1:1	1.13
BC_5_	40	48	88	1:1	0.73
BC_6_	127	119	246	1:1	0.26
BC_5_F_2_	211	75	286	3:1	0.23
BC_5_F_3_ (1)	641	227	868	3:1	0.61
BC_5_F_3_ (2)	588	205	793	3:1	0.31

### Genetic mapping of the *BnUC2* locus

Eight plants with up-curled leaves from the consecutive backcross BC_5_ family population were genotyped along with the two parents, NJAU5737 and ZS11, using a *Brassica* 60 K SNP bead chip array (Illumina, US). The SNP chip data analysis uncovered a BC_5_ plant (named BnUC2–5) with three segments, on chromosomes A05, C02, and C07, that differed from those in the recurrent parent ZS11 (Additional file 1: Table S1). The A05 differential segment contained 227 polymorphic SNP markers covering a 6.05-Mb interval between SNP markers M10447 and M11106. The C02 differential segment included 198 polymorphic SNP markers encompassing a 3.81-Mb interval between SNP markers M25019 and M36509, and the C07 differential segment harbored 231 polymorphic SNP markers covering an interval of 4.17 Mb between SNP markers M34819 and M44556. These three segments were possible candidate regions harboring the *BnUC2* locus. As other plants had larger genomic disparities than BnUC2–5, the latter plant was selfed and backcrossed to build populations for mapping of the up-curled leaf locus. As a result, 286 BC_5_F_2_ individuals and 246 BC_6_ individuals were obtained. Using these plants, 60 SSR-marker primer pairs were designed on the basis of the genomic sequences of the three differential segments. In SSR experiments, six polymorphic co-dominant SSR markers (BnaC02–12, BnaC02–14, BnaC07–04, BnaC07–05, BnaA05–21, and BnaA05–25) were found on the three segments.

All plants in BC_6_ and BC_5_F_2_ family populations were analyzed using the six polymorphic markers. Calculations of recombination frequencies in JoinMap 4.1 based on the resulting data suggested that the *BnUC2* locus was located on the A05 differential segment.

**Fig. 2:**
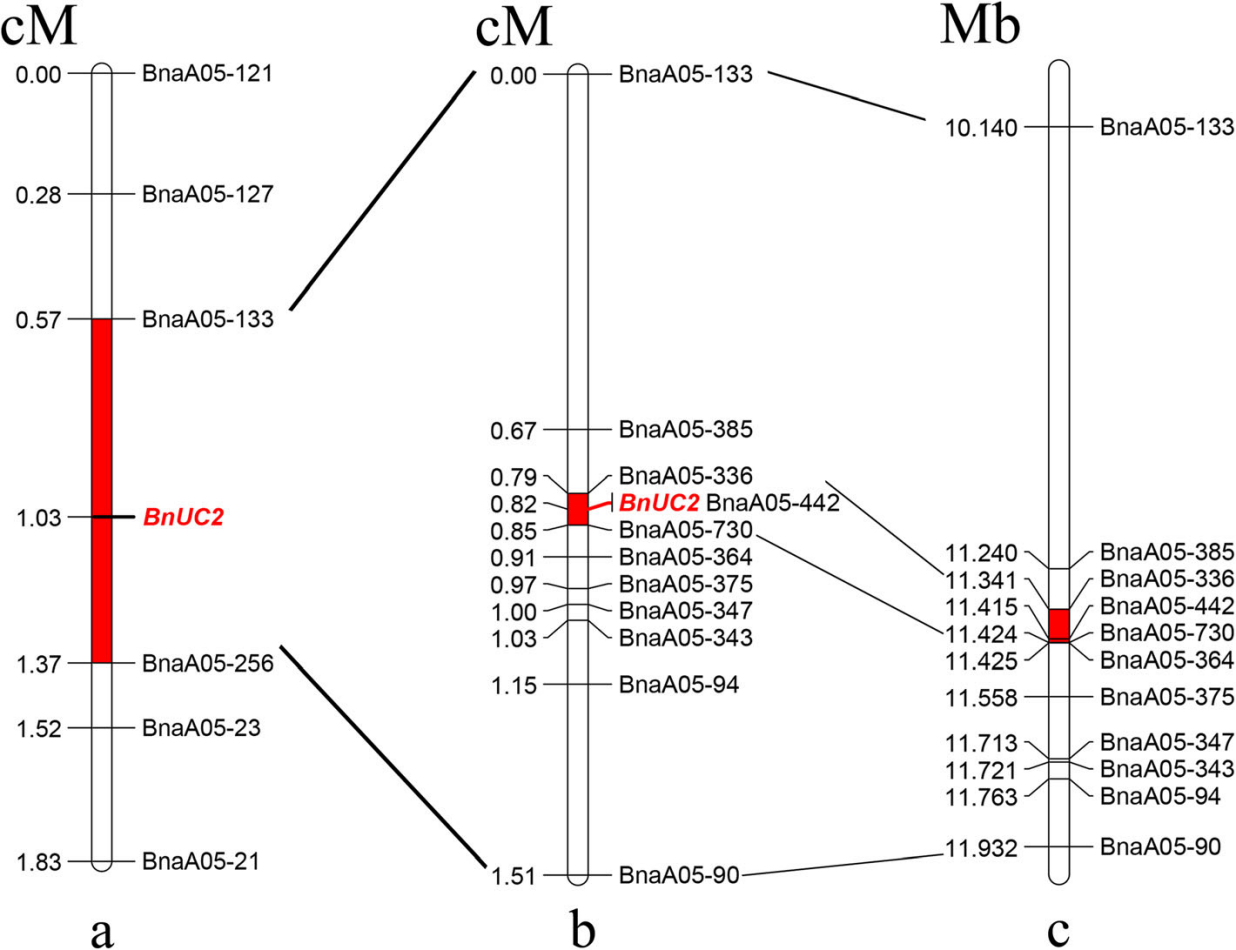
Genetic map and physical map of the *BnUC2* locus. **a**. The primary genetic map of *BnUC2* locus, the red indicates the mapping interval. **b**. Fine mapping of *BnUC2* locus. **c**. The physical map of *BnUC2* locus, the unit is Mb

Next, 70 SSR primer pairs were designed to map the *BnUC2* locus on the A05 chromosome. Five of these markers (BnaA05–121, BnaA05–127, BnaA05–133, BnaA05–256, and BnaA05–23) were found to be polymorphic (Additional file 2: Table S2). To construct linkage maps containing the up-curled-leaf trait locus *BnUC2* (Fig. 2a), 246 BC_6_ and 286 BC_5_F_2_ plants were genotyped with the five polymorphic markers. A linkage map including the *BnUC2* locus was then generated in JoinMap 4.1 and used to localize the *BnUC2* locus to a 3.84-Mb interval between SSR markers BnaA05–133 and BnaA05–256. The arrangement of the markers on the linkage map was in good agreement with the physical genome map of *B. napus*, thus indicating that this preliminary mapping was reliable.

**Fig. 3:**
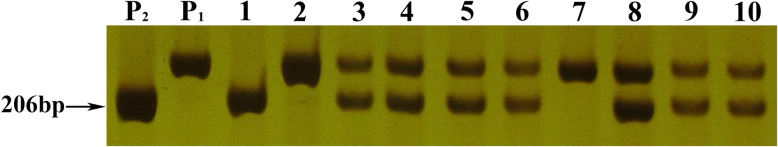
Experimental results of polymorphic marker BnaA05–442. Marker scan with progeny BC_5_F_3_ populations derived from the parents ZS11 and NJAU5737 was conducted. P_1_ and P_2_ indicates PCR products from the parents ZS11 and NJAU5737 plants, respectively. The number 3, 4, 5, 6, 8, 9 and 10 denote the PCR products from heterozygous plants with up-curled leaves, and 1 denotes the PCR products from homozygous plants with up-curled leaves, and 2 and 7 denote the PCR products from homozygous plants with flat leaves. Full-length, original blots image was presented in additional file 6: Fig. S3

To fine map the *BnUC2* locus, 1661 BC_5_F_3_ plants were obtained by selfing BC_5_F_2_ non-recombinant plants heterozygous at the *BnUC2* locus according to SSR marker analysis. In addition, we designed 160 SSR primers within the preliminary mapping interval, 10 of which were found to be polymorphic (BnaA05–385, BnaA05–336, BnaA05–442, BnaA05–730, BnaA05–364, BnaA05–375, BnaA05–347, BnaA05–343, BnaA05–94, and BnaA05–90) (Additional file 2: Table S2). Next, the 1661 BC_5_F_3_ plants were scanned with polymorphic markers, and linkage maps were obtained using JoinMap 4.1 (Fig. 2b). Finally, the *BnUC2* locus was mapped onto the interval between SSR markers BnaA05–730 and BnaA05–336, with marker BnaA05–442 found to be co-segregated with the *BnUC2* locus as shown by distinct SSR bands (Fig. 3). The frequency of recombination between BnaA05–730 and BnaA05–336 was 0.06%, and the mapping interval was 83.19 kb long. No other polymorphic marker was detected in the mapping interval. The order was completely consistent with the physical genome map of *B. napus* (Fig. 2c). These results verified the quality of the mapping.

### Effect of the *BnUC2* locus on plant height

To evaluate the effect of the *BnUC2* locus on plant height, all plants in the two BC_5_F_3_ family populations were genotyped with the co-dominant SSR marker BnaA05–442 co-segregating with *BnUC2,* and their heights were measured.

Analysis of variance (ANOVA) uncovered extremely significant variation with respect to the three marker-genotypes of BnaA05–442 co-segregating with leaf shape phenotype in the BC_5_F_3_ (1) and BC_5_F_3_ (2) populations (Table 4). This result clearly indicated that the *BnUC2* locus affects plant height.

**Table 4:** Analysis of variance of maker genotypes on plant heights in the BC_5_F_3_ (1) and BC_5_F_3_ (2) populations derived from NJAU5737 and the recurrent parent ZS11

Population	Source	*DF*	*SS*	*MS*	*F*	*F*_*0.01*_
BC_5_F_3_ (1)	Marker genotype	2	265,238.81	132,619.41	1152.85**	4.63
	Error	865	99,506.44	115.04		
	Total	867	364,745.25			
BC_5_F_3_ (2)	Marker genotype	2	231,933.09	115,966.54	939.77**	4.63
	Error	790	97,485.43	123.40		
	Total	792	329,418.51			

** indicates significant differences at 0.01 probability level

The effect of *BnUC2* genotype on average plant height was assessed in the BC_5_F_3_ (1) and BC_5_F_3_ (2) populations (Table 5). Compared with the heights of homozygous plants having normal, flat leaves, the average height of homozygous plants with up-curled leaves was reduced by 28.86 and 28.93% in BC_5_F_3_ (1) and BC_5_F_3_ (2) populations, respectively. These differences were extremely significant. Plant heights were also significantly lower in heterozygous plants with up-curled leaves, with reductions of 14.15 and 12.50% in BC_5_F_3_ (1) and BC_5_F_3_ (2) populations, respectively. We thus concluded that the *BnUC2* locus has a negative effect on plant height.

**Table 5:** Average plant heights of three marker-genotypes in the BC_5_F_3_ (1) and BC_5_F_3_ (2) populations derived from NJAU5737 and the recurrent parent ZS11

Population	Genotype	Sample size	Mean ± *SD* (cm)
BC_5_F_3_ (1)	Normal flat leaf	227	170.60 ± 11.95A
	Heterozygous up-curled leaf	430	146.46 ± 11.75B
	Homozygous up-curled leaf	211	121.36 ± 6.03C
BC_5_F_3_ (2)	Normal flat leaf	205	169.02 ± 12.45A
	Heterozygous up-curled leaf	405	147.89 ± 11.85B
	Homozygous up-curled leaf	183	120.12 ± 6.94C

Values in a column followed by different letters indicate significant differences by *LSD* (*P* = 0.01)

On the basis of these results, we concluded that the *BnUC2* locus not only controls leaf type, but also controls plant height, leading to a semi-dwarf phenotype. By regarding the *BnUC2* locus as a quantitative trait locus (QTL) for plant height, we were able to calculate its role. According to analysis of data from the BC_5_F_3_ (1) and BC_5_F_3_ (2) populations, the effect of the QTL was mainly additive (Table 5). The *BnUC2* locus explained 72.72 and 70.41% of phenotypic variation in plant height in the BC_5_F_3_ (1) and BC_5_F_3_ (2) populations, respectively. Frequency distributions in the two family populations were also analyzed (Additional file 3: Fig. S1). Based on polygene hypothesis in quantitative genetics [63], plant heights were not normally distributed but instead followed a multimodal distribution, consistent with the effect of a major gene. We thus concluded that the *BnUC2* locus is a major QTL for plant height.

### Candidate gene analysis

Using the *B. napus* Genome Browser (http://www.genoscope.cns.fr/brassicanapus/), five genes were identified and annotated in the fine mapping interval (Table 6). Two of these genes, *BnaA05g16680D* and *BnaA05g16700D*, are homologous to AT3G23050.1 and AT3G23030.1 found in the *Arabidopsis* Information Resource database that encode Aux/IAA7 and Aux/IAA2 proteins, respectively. These two *Arabidopsis* Aux/IAA proteins, which have been reported to act as repressors of auxin-regulated transcriptional activation, have four conserved domains (domains I, II, III, and IV) [41]. At high auxin concentrations, TIR1 interacts with Aux/IAA proteins via domain II to activate the degradation of Aux/IAA proteins by 26S proteasome, with ARFs then released [64]. Mutations of Aux/IAA family genes can lead to defective plant growth and development, including the generation of shorter hypocotyls and stature, leaf curling, reduction in lateral root number, and loss of apical dominance [17, 47, 48, 65–67]. *BnaA05g16680D* and *BnaA05g16700D* were thus considered to be candidate genes responsible for leaf up-curling and plant semi-dwarfing.

**Table 6:** Function annotation of genes in the mapping interval

Gene in *B. napus*	Chromosome position	Homologue in *A. thaliana*	Gene annotations
*BnaA05g16680D*	11341560-11342921	*AT3G23050.1*	Aux/IAA7 protein
*BnaA05g16690D*	11375956-11376874		unknown protein
*BnaA05g16700D*	11384467-11385470	*AT3G23030.1*	Aux/IAA2 protein
*BnaA05g16710D*	11398936-11401491	*AT3G23020.1*	Tetratricopeptide repeat (TPR)-like superfamily protein
*BnaA05g16720D*	11401616-11403042	*AT3G23000.1*	CBL-interacting protein kinase 7

*BnaA05g16690D* in the mapping interval encodes an unknown protein, while *BnaA05g16710D* is homologous to *AT3G23020.1*, which encodes a tetratricopeptide repeat (TPR)-like superfamily protein. *BnaA05g16690D* and *BnaA05g16710D* have no previously reported relationship to leaf type and plant height regulation.

*BnaA05g16720D* is homologous to *AT3G23000.1*, which encodes a CBL interaction protein kinase 7 (CIPK7). CIPK7 is involved in the regulation of plant adversity stress and participates in plant signal transduction during response to abiotic stress conditions [68, 69]. No association has been reported between *BnaA05g16720D* and the regulation of leaf type and plant height.

**Fig. 4:**
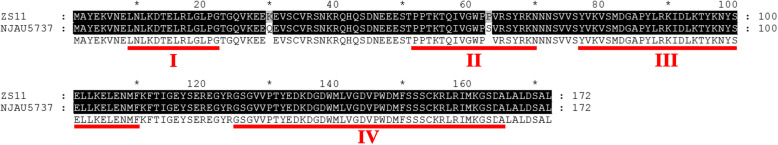
Amino acid sequence alignment of *BnaA05g16700D* (*BnaA05.IAA2*) in ZS11 and NJAU5737. Four conserved domain of *BnaA05g16700D* were designated as I, II, III and IV, indicated by red underline

To further assess the above gene candidates for the *BnUC2* locus, we performed comparative sequencing of NJAU5737 and ZS11, the parents of the mapping populations. The DNA sequences of *BnaA05g16680D*, *BnaA05g16690D*, and *BnaA05g16710D* were identical between the two parents, but differences were found in *BnaA05g16700D* and *BnaA05g16720D*. Two single-nucleotide transition mutations, leading to two amino-acid substitutions at positions 30 and 63 (Fig. 4), were present in *BnaA05g16700D* (named *BnaA05.IAA2*) of NJAU5737. The substitution at amino-acid position 63 was located in the Degron motif (GWPPV) of domain II, which is strongly conserved in most plant IAA family members. *BnaA05g16700D* is thus the gene most likely corresponding to the *BnUC2* locus. We then developed a pair of site specific CAPS marker (Additional file 4: Table S3) based on the variant site on the Degron motif (GWPPV) of BnaA05.IAA2, which completely co-segregated with the phenotypes in the BC_5_F_3_ population (Additional file 5: Fig. S2). These results demonstrated that *BnaA05g16700D* is thus the gene most likely corresponding to the *BnUC2* locus.

### Agronomic traits

To evaluate the effect of the *BnUC2* locus on plant agronomic traits, 20 plants with up-curled leaves and 20 with flat leaves were randomly sampled from the BC_6_ population derived from NJAU5737 and recurrent parent ZS11. Plant height, branching height, main inflorescence length, and silique length were significantly smaller in up-curled-leaf plants than in flat-leaf plants, with no alterations observed in other analyzed traits (Table 7). These results indicate that the *BnUC2* locus significantly reduces plant height, branching height, and main inflorescence length but has no significant influence on yield.

**Table 7:** Agronomic traits of flat leaf and up-curled leaf plants in the BC_6_ derived from NJAU5737 and the recurrent parent ZS11

Trait	Flat leaf plants	Up-curled leaf plants
Plant height (cm)	175.64 ± 13.67	149. 64 ± 13.99^a^
Branching height (cm)	61.75 ± 9.57	54.53 ± 9.55^b^
Main inflorescence length (cm)	71.29 ± 8.33	54.64 ± 8.82^a^
Number of first effective branch	6.90 ± 1.45	6.20 ± 1.14
Stem diameter (mm)	20.38 ± 3.33	18.22 ± 3.20
Number of siliques on the main inflorescence	77.5 ± 11.1	70.3 ± 8.6
Total siliques per plant	395.4 ± 89.3	351.6 ± 95.1
Silique length (cm)	10.11 ± 0.97	9.43 ± 1.09^b^
Seeds per siliques	25.38 ± 3.74	26.46 ± 3.09
1000-seed weight (g)	4.75 ± 0.14	4.54 ± 0.11
Yield per plant (g)	24.61 ± 11.68	21.08 ± 9.35

^a^ and ^b^ indicate significant differences at 0.01 and 0.05 probability level by *t*-test, respectively. Data are shown as mean ± *SD*, *n* = 20

## Discussion

A plant type with upright leaves and a short stature is undoubtedly beneficial for the improvement of crop yield and lodging resistance [2]. In *B. napus*, the mechanisms underlying plant height are complex, and many plant height loci/QTLs have been reported [17, 51, 58, 59, 61, 70–74]. No technical breakthroughs have yet been achieved in the breeding of dwarf stature canola variety, however, and further mining and utilization of new dwarf or semi-dwarf germplasm resources is thus required. In the present study, we characterized a novel germplasm resource with up-curled leaves and a semi-dwarf stature. We identified a locus, *BnUC2*, in this germplasm resource that exhibited dominance in the populations but had mainly additive effects on plant height and explained 72.72 and 70.41% of phenotypic variation in plant height in the two BC_5_F_3_ family populations. These results suggest that the up-curled leaf trait of the *Bnuc2* can serve as an indicator trait for semi-dwarf variety breeding. Meanwhile, it can be useful for increasing planting density, with the aim of elevating *B. napus* yield. In addition, heterozygous *BnUC2* locus does not have any significant effect on yield per plant. Thus, the newly characterized germplasm resource harboring the *BnUC2* locus may probably be an aid in *Brassica* variety breeding.

Our primary mapping of *BnUC2* positioned this locus within a 3.84-Mb interval, which corresponds to a recombination rate of 0.8% based on the linkage map. This value is far larger than the *B. napus* genome-level average of 400 kb/cM [75], this is most likely because the *BnUC2* locus is located near the chromosome A05 centromere that affects chromosomal recombination. This may also be caused by the limited number of plants used for primary mapping. To fine map the *BnUC2* locus, we expanded the mapping population and 1661 BC_5_F_3_ plants were obtained, leading to a fine-mapping interval of 83.19-kb in length, with a corresponding recombination rate of 0.06%.

On the basis of our mapping, gene sequencing, and bioinformatics analysis results, *BnaA05.IAA2* is the gene most likely responsible for leaf up-curling and plant semi-dwarfing. This gene encodes an AUX/IAA2 protein that acts as a repressor of auxin-regulated transcriptional activation. Two single amino-acid changes were identified in the AUX/IAA2 protein. The substitution at amino-acid position 63, located in the domain-II Degron motif (GWPPV) conserved in most plants, is key to the phenotypic mutation [41]. Auxin, one of the most important hormones in plants [76, 77], is involved in the regulation of multiple traits, including leaf type and plant height [34, 47, 65, 78]. Mutations of genes related to auxin synthesis and signal transduction may lead to leaf curling and dwarf/semi-dwarf phenotypes. *BnaA05.IAA2* is thus the reliable candidate gene and has a pleiotropic effect on leaf type and plant height. Another candidate gene, *BnaA05g16720D*, with coding sequence mutations related to stress response [68, 69], could not be excluded completely.

## Conclusions

A novel pure accession with up-curled leaves and a semi-dwarf stature found in oilseed rape breeding was used for investigation of the mutated leaf and plant type trait. Results showed that the up-curled leaf trait was controlled by a dominant locus (*BnUC2*) fine-mapped onto an 83.19-kb interval on chromosome A05 using SNP and SSR markers. Our results have also been found that *BnUC2* is a major plant height QTL responsible for the observed semi-dwarf stature. Comparative sequencing shows that *BnaA05g16700D* (*BnaA05.IAA2*) and *BnaA05g16720D* in the fine mapping interval which contains five genes, are mutated in NJAU5737. A substitution (P63S) of *BnaA05.IAA2* in NJAU5737 found in the conserved Degron motif GWPPV completely co-segregated with the phenotypes as demonstrated by the specific CAPS marker experiments. This mutation is of functionality, may probably result in the phenotype of up-curled leaves and a semi-dwarf stature. Our findings may provide an effective foundation for the semi-dwarf variety breeding of *B. napus* and exploration of the leaf up-curling and dwarfing mechanism.

## Methods

### Plant materials and analysis of inheritance of the up-curled leaf trait

The *Bnuc2* mutant exhibiting leaf up-curling and a semi-dwarf stature was derived from a pure canola line, NJAU5737. NJAU5737 was crossed with canola variety ZS11 to produce the F_1_ generation. The F_1_ individuals were then backcrossed with ZS11 to produce progeny populations. The self and backcross populations were examined to determine the segregation ratio of plants with up-curled vs. flat leaves. Chi-square tests were performed to test the genetic regulation of the up-curled leaf trait. BC_5_, BC_6_, and BC_5_F_2_ family populations derived from NJAU5737 and ZS11 were used for preliminary mapping of the up-curled leaf trait locus *BnUC2*, and the BC_5_F_3_ family population was used for fine mapping the *BnUC2* locus.

All materials were grown in fields at the Jiangpu Experimental Station of Nanjing Agricultural University (Jiangsu Province, China). Plants were sown uniformly in 2.5-m long rows, with 0.4 m between rows and 15 individuals per row.

### SNP analysis

Eight BC_5_ plants with up-curled leaves and the two parents (ZS11 and NJAU5737) were selected for SNP genotyping using a *Brassica* 60 K SNP bead chip array (Illumina, US). The genotyping was performed to detect chromosome segments differing between the backcross progeny population and ZS11, thereby preliminarily identifying the chromosome on which the *BnUC2* locus was located. The SNP marker was named using “M” plus an index number specified by Genome Studio v2011.1 (Illumina, US). The SNP analysis was identical to that of a previous study [61].

SNPs on 19 chromosomes were compared between the eight up-curled-leaf plants and the recurrent parent ZS11 to determine differential chromosome segments. The BC_5_ plant having the fewest genome-level differences with ZS11, BnUC2–5, possessed three differential segments located on chromosomes A05, C02, and C07.

### Genetic mapping of the *BnUC2* locus

BnUC2–5 was selfed and backcrossed with ZS11, and 286 BC_5_F_2_ and 246 BC_6_ individuals were obtained for mapping the *BnUC2* locus, respectively.

SSR-marker primers were designed in Primer Premier 5.0 [79] using the genomic sequences of the three differential fragments downloaded from the *B. napus* Genome Browser. SSR markers that were polymorphic between BnUC2–5, ZS11, and NJAU5737 were used to detect polymorphisms in all plants in BC_6_ and BC_5_F_2_ family populations. Recombination frequencies between SSR markers and the *BnUC2* locus were calculated using JoinMap 4.1 software to determine the chromosome harboring the *BnUC2* locus.

For fine mapping of the *BnUC2* locus, we used 1661 BC_5_F_3_ plants obtained from non-recombinant BC_5_F_2_ plants. On the basis of preliminary mapping results, polymorphic SSR markers were gradually developed, and a genetic map was constructed in JoinMap 4.1 to narrow the interval containing the *BnUC2* locus.

Polymerase chain reaction (PCR) amplifications of molecular markers were performed as described previously [54]. Total DNA extraction and linkage map construction were carried out according to previous reports [61].

### Identification of genes in the mapping interval and comparative sequencing

To identify genes in the mapping interval, the genomic sequence of the mapping interval carrying the *BnUC2* locus was downloaded from the *B. napus* Genome Browser. Genes detected in the mapping interval were annotated and then cloned. Gene-amplification primers based on the genomic sequence were designed using Primer Premier 5.0 (Additional file 4: Table S3). Full-length sequences of genes in the mapping interval were amplified using genomic DNA and cDNA from parents NJAU5737 and ZS11.

PCR amplifications were performed in 50-μL reaction volumes using PrimeSTAR Max DNA polymerase (Takara, Tokyo, Japan). The PCR conditions were as follows: 94 °C for 2 min, followed by 35 cycles of 98 °C for 10 s, annealing for 5 s at the annealing temperature of each gene-amplification primer, and 72 °C for 30s, with a final extension of 72 °C for 5 min.

The PCR products were purified using a AxyPrep DNA Gel Extraction kit and sequenced by GenScript Biotech (Nanjing, China). The resulting sequences were aligned using Clustal X and GeneDoc software.

### Development of site specific CAPS marker

Based on the comparative sequencing, nucleotides at positions 184–187 were CCTC in parent ZS11, and has been mutated to CCTT in parent NJAU5737. To detect this single-nucleotide transition mutation, specific CAPS primers (Additional file 4: Table S3) were designed to PCR amplifications. The PCR products were 186 bp long and then were digested using MnlI (Catalog R0163S, New England Biolabs, recognition site: CCTC). The PCR products containing the CCTC recognition site were cut into two segments, 127 bp and 59 bp in length, respectively. Then the polymorphic bands were separated by polyacrylamide gel electrophoresis.

### Agronomic traits

We randomly selected 20 up-curled-leaf and 20 flat-leaf plants from the BC_6_ population to investigate traits for evaluation of the effect of heterozygous *BnUC2* locus on plant agronomic efficiency. The agronomic trait data included plant height, branching height, main inflorescence length, number of first effective branch, stem diameter, number of siliques on the main inflorescence, total siliques per plant, silique length, seeds per siliques, 1000-seed weight, and yield per plant. The mean values of all agronomic traits between up-curled-leaf and flat-leaf plants were compared by *t*-tests.

Heights of all plants in two BC_5_F_3_ family populations were determined, and an ANOVA was applied to estimate the genetic variance, error variance, and phenotypic variation of this trait.

### Determination of chlorophyll content and photosynthetic efficiency

We randomly selected 15 homozygous up-curled-leaf and 15 flat-leaf plants from the BC_5_F_3_ population at the seedling stage for determination of chlorophyll content. Chlorophyll (Chl) was extracted from 0.2-g fresh leaves with 50 ml of 80% acetone, and Chl contents were determined using an Alpha-1500 spectrophotometer (LASPEC, Shanghai, China). The leaf Chl a, Chl b, and total Chl contents were measured as described previously [80].

Six homozygous up-curled-leaf and six flat-leaf plants were selected randomly from the BC_5_F_3_ population at the seedling stage for determination of photosynthetic efficiency. The photosynthetic characteristics were determined using a Li-Cor 6400 portable photosynthesis system (Li-Cor Inc., Lincoln, NE, USA) at 23 °C as described previously [54]. All data were collected between 09:00 am and 11:00 am.

## Supplementary information

**Additional file 1 **: **Table S1**. Distribution of polymorphic SNP marker between *Bnuc2* and ZS11 on each chromosome.

**Additional file 2 **: **Table S2**. The designed SSR markers used in this study.

**Additional file 3 **: **Figure S1**. Frequency distribution of plant height in the BC_5_F_3_ (1) and BC_5_F_3_ (2) populations.

**Additional file 4 **: **Table S3**. The designed primers of comparative sequencing and CAPS marker used in this study.

**Additional file 5 **: **Figure S2**. Part of marker experimental results for CAPS marker.

**Additional file 6 **: **Figure S3**. Full-length, original blots image of Fig. 3.

## Data Availability

The datasets generated and/or analysed during the current study are available in the Figshare repository (10.6084/m9.figshare.12689615).

## Abbreviations

*B. napus*: *Brassica napus*
*BnUC2*: Up-curled leaf locus in *B. napus* genome
*Bnuc2*: Up-curled leaf mutant
Chl: Chlorophyll
PCR: Polymerase chain reaction
TIR1/AFB: Transport inhibitor resistant1/auxin signaling F-box
ARF: Auxin response factor
SNP: Single nucleotide polymorphism
SSR: Simple sequence repeat
TAIR: The *Arabidopsis* Information Resource
ZS11: Zhongshuang 11
ANOVA: Analysis of variance
QTL: Quantitative Trait Locus
HD-ZIP III: Class III HOMEODOMAIN LEUCINE-ZIPPER
WOX: WUSCHEL RELATED HOMEOBOX
TCP: TB1-CYC-PCFs
Aux/IAA: AUXIN/INDOLE-3-ACETIC ACID
