# Supplementary material for: Fine mapping of the BnUC2 locus related to leaf up-curling and plant semi-dwarfing in Brassica napus
Source: BMC Genomics. 2020 Jul 31;21:530. doi: 10.1186/s12864-020-06947-7 (PMC7430850; doi:10.1186/s12864-020-06947-7)
Supplement: Supplementary file 1 — Additional file 1 : Table S1. Distribution of polymorphic SNP marker between Bnuc2 and ZS11 on each chromosome. [file 12864_2020_6947_MOESM1_ESM.docx]

**Additional file 1: Table S1** Distribution of polymorphic SNP marker between *Bnuc2* and ZS11 on each chromosome

| Chromosome | Difference SNPs No. | Max adjacent No. |
| --- | --- | --- |
| A01 | 34 | 26 |
| A02 | 9 | 1 |
| A03 | 5 | 2 |
| A04 | 68 | 25 |
| A05 | 457 | 227 |
| A06 | 52 | 21 |
| A07 | 86 | 74 |
| A08 | 14 | 5 |
| A09 | 2 | 1 |
| A10 | 202 | 78 |
| C01 | 29 | 15 |
| C02 | 435 | 198 |
| C03 | 128 | 77 |
| C04 | 66 | 33 |
| C05 | 24 | 5 |
| C06 | 191 | 65 |
| C07 | 413 | 231 |
| C08 | 3 | 1 |
| C09 | 58 | 27 |
| Total | 2280 | 1081 |
