# Supplementary material for: Fine mapping of the BnUC2 locus related to leaf up-curling and plant semi-dwarfing in Brassica napus
Source: BMC Genomics. 2020 Jul 31;21:530. doi: 10.1186/s12864-020-06947-7 (PMC7430850; doi:10.1186/s12864-020-06947-7)
Supplement: Supplementary file 2 — Additional file 2 : Table S2. The designed SSR markers used in this study. [file 12864_2020_6947_MOESM2_ESM.docx]

**Additional file 2: Table S2** The designed SSR markers used in this study.

| Name of primers | Sequence of primers | Product length | Chromosome location |
| --- | --- | --- | --- |
| BnaC02-01-F | ATCGCCTCCTATTTCTCG | 223 | 40014287 |
| BnaC02-01-R | CTTCCTTCTGTGCCGCTA |  |  |
| BnaC02-02-F | GCTCCGTTTTCCCGTTAT | 208 | 40204800 |
| BnaC02-02-R | ATGTTCCGCCATTGATGT |  |  |
| BnaC02-03-F | AGAGTCAACCCTTGTCCTT | 231 | 40425729 |
| BnaC02-03-R | TTTACCTGGAAACCTTGTG |  |  |
| BnaC02-04-F | GATGATGAAAGTGAAAAGGAA | 207 | 40511972 |
| BnaC02-04-R | TCTAGAGGCAAGAAAACCC |  |  |
| BnaC02-05-F | AAACTGCGGCAACCAAAC | 172 | 40648371 |
| BnaC02-05-R | CTCCTTACAACGCCAACA |  |  |
| BnaC02-06-F | TCATGTTCAGACTCCTCCTA | 260 | 40451804 |
| BnaC02-06-R | CACTTGCCTTGATTGTTT |  |  |
| BnaC02-07-F | ATCCAGAAATAGGAAACATG | 235 | 40805054 |
| BnaC02-07-R | CTAACGAGGGAAATAGAGG |  |  |
| BnaC02-08-F | TTCCTGAGCGATGAAAGT | 219 | 40246782 |
| BnaC02-08-R | TCAAGGCAATGCGTAGAG |  |  |
| BnaC02-09-F | TGGGCTAGGATTAACGAG | 193 | 40259387 |
| BnaC02-09-R | TGGAGCACCATTTCTTGT |  |  |
| BnaC02-10-F | ACATTGTGGCGTCCTTAG | 199 | 40313153 |
| BnaC02-10-R | CCATTGTTATCACGGTTG |  |  |
| BnaC02-11-F | GGGAAATAAGTGACGAGGA | 226 | 40409478 |
| BnaC02-11-R | TGGCAACTGAGGATGAAG |  |  |
| BnaC02-12-F | TGTGATCATAATGTCTCGGA | 180 | 40704719 |
| BnaC02-12-R | GGTTGTGGTTTCTGGTTG |  |  |
| BnaC02-13-F | AGGTTTAGGTGGTGGTCT | 246 | 40456005 |
| BnaC02-13-R | TGCGGAGTGTAGTGAAGT |  |  |
| BnaC02-14-F | GCGTATTGACTTCCTGTTT | 269 | 40621891 |
| BnaC02-14-R | TTCTTCGCTTTCTTCTGG |  |  |
| BnaC02-15-F | ACAATTCCAGAGCCCTAA | 190 | 40554820 |
| BnaC02-15-R | TTCGCTTTCTCGTTCATC |  |  |
| BnaC02-16-F | CGAGTCATCCTTGAAATC | 186 | 40590505 |
| BnaC02-16-R | TACGGAAAATATGGTGGT |  |  |
| BnaC02-17-F | ATTCTCAGTGACTATGCGATTC | 230 | 40613053 |
| BnaC02-17-R | AAGGCTGTCCCTCCCTCT |  |  |
| BnaC02-18-F | GCAGGAGGAAGAGGAGTC | 227 | 40510722 |
| BnaC02-18-R | GATGTATCAAAGCGAGGA |  |  |
| BnaC02-19-F | TCATCGGCTTCCTCCATC | 191 | 40665187 |
| BnaC02-19-R | CGTCAAACAGGTCCTTCGT |  |  |
| BnaC02-20-F | CATCATGGCTGTACCTTT | 263 | 40770643 |
| BnaC02-20-R | ATGGCTTCTGAGATTCGT |  |  |
| BnaC07-01-F | AGATTTCGGTTCGGGTTT | 171 | 5349562 |
| BnaC07-01-R | TGGCTACGCTAATGTTCG |  |  |
| BnaC07-02-F | CCCGTTGTTGCTGTATGG | 173 | 5828111 |
| BnaC07-02-R | AAGATGCAGAAGCCGTAG |  |  |
| BnaC07-03-F | ACCCTTCTGATTTGATTTTG | 178 | 5327918 |
| BnaC07-03-R | ACAGTGTTGTTCGATTTCCT |  |  |
| BnaC07-04-F | AAAATATGTTAGACGTAGATTG | 182 | 6082025 |
| BnaC07-04-R | ATGAGTGATGCAGAAAAGA |  |  |
| BnaC07-05-F | ACCACCTAGCCAACAAAT | 184 | 5691109 |
| BnaC07-05-R | CAGAGCCACTCAACCAAT |  |  |
| BnaC07-06-F | CATCTTCCAGTGAATCGT | 190 | 5639324 |
| BnaC07-06-R | ATTTAGTGGGACCTGATT |  |  |
| BnaC07-07-F | TCACCAGGTTCCACAACA | 193 | 5541906 |
| BnaC07-07-R | TCCAGTGAAATGCTCCAA |  |  |
| BnaC07-08-F | TCCTGACTTCCCGCACAT | 197 | 5866372 |
| BnaC07-08-R | TGGTCTCGTCGTCGCTCT |  |  |
| BnaC07-09-F | ATTGCGAGTCCACGTATTA | 199 | 5821446 |
| BnaC07-09-R | CGAGGAAGAAACGAAAGTC |  |  |
| BnaC07-10-F | CTTCGCTGGTCTTCCCTC | 208 | 5802727 |
| BnaC07-10-R | AGCAAGTTCAAGCCCTGT |  |  |
| BnaC07-11-F | AACTTTGCTGTGGGCTATG | 209 | 5575457 |
| BnaC07-11-R | CGGCTCGGAGGTTCTTTA |  |  |
| BnaC07-12-F | GAGGGTAGAGCAGAGGAA | 210 | 5624257 |
| BnaC07-12-R | CTTGAGTAGGTGGTGGTG |  |  |
| BnaC07-13-F | CTATCTTGCTTGACTGCG | 218 | 5970877 |
| BnaC07-13-R | GCTGACCTCCAACCTAAC |  |  |
| BnaC07-14-F | GATTCTTGGCAACTTCTG | 220 | 5718830 |
| BnaC07-14-R | CTCACCTCTTCCACCTTC |  |  |
| BnaC07-15-F | CGGGATCGTCCAAGACAT | 223 | 5539444 |
| BnaC07-15-R | CGTTCCGTGGGTGAGGTA |  |  |
| BnaC07-16-F | AATCACAAATGCCTACAAAC | 246 | 5577871 |
| BnaC07-16-R | CTATTTCAGACTTTCGGTTT |  |  |
| BnaC07-17-F | ATTTGATTTACCTAACCCAC | 261 | 5300636 |
| BnaC07-17-R | GGATATTCGCCAACCTAC |  |  |
| BnaC07-18-F | CGGTTGATTGAAATGATT | 278 | 5540699 |
| BnaC07-18-R | AGCAAACACGGTGTCTAT |  |  |
| BnaC07-19-F | AAGACGACGACAACGAAG | 281 | 6144693 |
| BnaC07-19-R | CAAGATCAGGAGGAGTGG |  |  |
| BnaC07-20-F | ATTACAATCCCTGTATGGTT | 282 | 5762741 |
| BnaC07-20-R | AATGAATAAGTGTTCCCTTT |  |  |
| BnaA05-06-F | CGAAACTCACGACTGGATAA | 175 | 11359525 |
| BnaA05-06-R | ATGGGTGGTGATGATGTTG |  |  |
| BnaA05-07-F | AATTTTAACATACACTTGGATC | 180 | 11350530 |
| BnaA05-07-R | TTGATTATGTCCTTAACATTCT |  |  |
| BnaA05-08-F | ATGTTGAAAAGTTTTGTGCGT | 184 | 11349838 |
| BnaA05-08-R | CTATCGGCTGATCCGGTC |  |  |
| BnaA05-09-F | CTGTATTTCCTATCGGTTTC | 202 | 11348213 |
| BnaA05-09-R | ATTAGTTCTTTGGTCTTATTGT |  |  |
| BnaA05-10-F | AGTTGATCTTTCCTAACACTTTT | 208 | 11359609 |
| BnaA05-10-R | AGGTGACTGCGGTTTGAG |  |  |
| BnaA05-11-F | AGAGGAGGATGATTTTGATA | 209 | 11351121 |
| BnaA05-11-R | ATTAGGTTTTAGACTTTTAGATAG |  |  |
| BnaA05-12-F | GCTAATCTTATTGGGACTCC | 213 | 11349731 |
| BnaA05-12-R | GGTATTAGAGCAAAGATGATGA |  |  |
| BnaA05-13-F | TTTAACATAGCTTATTACGGAC | 229 | 11360292 |
| BnaA05-13-R | AAAACTATAGGTGGTTTGAATA |  |  |
| BnaA05-14-F | GTTAGGTCTTACAAAACAGTG | 235 | 11353308 |
| BnaA05-14-R | CATGAGTATCCAAAAGTACATA |  |  |
| BnaA05-15-F | GGCCTAATAATGAACTTGAA | 238 | 11360557 |
| BnaA05-15-R | GATGATTGGTTGGTTGATAC |  |  |
| BnaA05-16-F | ATCCGAATACCCGAAATA | 243 | 11348018 |
| BnaA05-16-R | AATACAGGTTCAGGTCCGT |  |  |
| BnaA05-17-F | AGAAGTCTTTTCCCTCTTTAA | 254 | 11348617 |
| BnaA05-17-R | GTTTTCATTTGGAGATTTTG |  |  |
| BnaA05-18-F | AATTGATTTGCTTGCAGAAG | 258 | 11361009 |
| BnaA05-18-R | ATGCCATATTATGAAAGTCCA |  |  |
| BnaA05-19-F | CTCCAAACCCACTCTAATCTC | 259 | 11356299 |
| BnaA05-19-R | ATATCAAACGCAAATCTGAAA |  |  |
| BnaA05-20-F | AGAAGTCTTTTCCCTCTTT | 261 | 11348617 |
| BnaA05-20-R | GATCAATGTTTTCATTTGG |  |  |
| BnaA05-21-F | TCTCAAGTGGTTGTCTCA | 208 | 15336447 |
| BnaA05-21-R | TCATGGTTATTTTCTCGT |  |  |
| BnaA05-22-F | TTATCCGAATACCCGAAAT | 268 | 11347774 |
| BnaA05-22-R | AAAGTGAACCGAAACCGA |  |  |
| BnaA05-23-F | GCTTCTTAATGAAACTTGAAAGG | 225 | 14900487 |
| BnaA05-23-R | TATCATATTTTTAGGGTGATTG |  |  |
| BnaA05-24-F | AACATAACATTGGAAAATTGCA | 161 | 11341236 |
| BnaA05-24-R | GGGACTTATTTGTCGAATAACC |  |  |
| BnaA05-25-F | TGGTGAGAATTGTGAAAGAGGGA | 281 | 15170180 |
| BnaA05-25-R | AGGAGCGGCAGAGGAAAGC |  |  |
| BnaA05-26-F | CTCCAAAACATTCGTGACA | 175 | 11341537 |
| BnaA05-26-R | GTGTCATATTTATTACCCAACA |  |  |
| BnaA05-27-F | AGCGGATGGAGTTGTAGGAG | 177 | 11338145 |
| BnaA05-27-R | CACCCGAAAACTTTGAGGC |  |  |
| BnaA05-28-F | TCACCCACGAAACCATTT | 185 | 11331303 |
| BnaA05-28-R | GAGGTCTCCATTATCCGAGT |  |  |
| BnaA05-30-F | CAGTTCTGGAGCATTTAGGA | 210 | 11337259 |
| BnaA05-30-R | ATTGGGTAGAGGCGTGTAT |  |  |
| BnaA05-31-F | AGCTCCTTTCTGATACACCTC | 212 | 11340450 |
| BnaA05-31-R | AACCTAGAATCTTTCCCTCAC |  |  |
| BnaA05-32-F | AGAAGCAGGGTTCCGAGG | 212 | 11341258 |
| BnaA05-32-R | GTGCAATTTTCCAATGTTATGTT |  |  |
| BnaA05-34-F | ATCCAGACGAAAGAGGAACATG | 230 | 11340788 |
| BnaA05-34-R | TCTCCCTGACCCACCCGA |  |  |
| BnaA05-35-F | TATCATCCTTCAAGTGCTAAA | 241 | 11332097 |
| BnaA05-35-R | AATTGTTCTAACCCTAGTCCA |  |  |
| BnaA05-36-F | TTGTCCATTCTTGATTTCCCTT | 277 | 11339735 |
| BnaA05-36-R | CCGCTCCCAAACACCATT |  |  |
| BnaA05-37-F | CTAGTGGTGACTGATCATCGAC | 278 | 11341482 |
| BnaA05-37-R | ATGGAGAAGTACTGCAAGAACA |  |  |
| BnaA05-38-F | GTGAGTGAATGGAATTACGG | 281 | 11333037 |
| BnaA05-38-R | TTGCAGTTTTGACGGAAA |  |  |
| BnaA05-39-F | CGCTTCCTCAATACCTCTA | 295 | 11338075 |
| BnaA05-39-R | AAACTTCCTTACCACCCTC |  |  |
| BnaA05-41-F | ATCCATGCGATTTGTCGA | 172 | 11333056 |
| BnaA05-41-R | CCGTAATTCCATTCACTCACT |  |  |
| BnaA05-86-F | TGACAGAAGGTGAAACGAA | 170 | 11977640 |
| BnaA05-86-R | TGAATTAAACCGAGCAGAC |  |  |
| BnaA05-88-F | GGCGACCTGACTTCCTCTG | 175 | 11863342 |
| BnaA05-88-R | GCATCACCGACCATCATTTA |  |  |
| BnaA05-89-F | ATCAGCTTCCCTACGTTTC | 177 | 11961253 |
| BnaA05-89-R | CATTCGCATCAGATTTGTG |  |  |
| BnaA05-90-F | GGTGGATGTTTGAAGATTTT | 192 | 11931716 |
| BnaA05-90-R | AGGACCTAAGGACTTAAGCA |  |  |
| BnaA05-91-F | ATTGACGCCAAGACTCCTA | 205 | 11945360 |
| BnaA05-91-R | ATTGACAGCGACCTTGATA |  |  |
| BnaA05-92-F | TCTCGCTTCTCATCATTCTA | 205 | 11886902 |
| BnaA05-92-R | ACATCTGCTTCGGACCTT |  |  |
| BnaA05-93-F | AGCAAGACTGTCCAACTCG | 210 | 11619006 |
| BnaA05-93-R | GAAGCAATCAGCAAGAAGA |  |  |
| BnaA05-94-F | GACCACGAGGGCTTGTAT | 213 | 11763318 |
| BnaA05-94-R | TGCAGTGCAGTTGTTTCC |  |  |
| BnaA05-95-F | CGGGATCGGAGCTAGGTTA | 222 | 11911336 |
| BnaA05-95-R | AGGATGGTCGGAGGCAATA |  |  |
| BnaA05-97-F | AACATGAGCGGTCAAATAA | 265 | 11814545 |
| BnaA05-97-R | GTCTAATGCTTCCAGTGCC |  |  |
| BnaA05-98-F | TAATGCAAACATATTCAACG | 284 | 11698681 |
| BnaA05-98-R | GATGGAAAGAACATACCGA |  |  |
| BnaA05-99-F | GGTTTTGTCTCCAGCGTC | 300 | 11521329 |
| BnaA05-99-R | CATGCACCACATACATCTTTA |  |  |
| BnaA05-100-F | TGTGGATTATGACGGTTTA | 174 | 12212574 |
| BnaA05-100-R | AGGTCGGTTTTAGCTTTT |  |  |
| BnaA05-101-F | TCACTGCCTTCCCATTCT | 183 | 12413915 |
| BnaA05-101-R | ACGGTGGGGTAAACTCTG |  |  |
| BnaA05-102-F | TCATCGCTTCCGTCTTAG | 183 | 12366411 |
| BnaA05-102-R | GGTGACTTGTAGCCTTTGC |  |  |
| BnaA05-103-F | CCACATTTTCCCTAACTCG | 185 | 12547720 |
| BnaA05-103-R | CTCTGCCACCTTGTCTCAC |  |  |
| BnaA05-104-F | ACAGAGCCCAGAGGAACAG | 188 | 12341914 |
| BnaA05-104-R | CATCGTCACTCCCTACAGC |  |  |
| BnaA05-105-F | ATCAAGCCGTAGCAAGGT | 210 | 12700227 |
| BnaA05-105-R | TCCGCTTCAGCAACAATA |  |  |
| BnaA05-106-F | TTTTCTAAATCAAATCCGA | 224 | 12451108 |
| BnaA05-106-R | GCACTCACTTGTCCTGTT |  |  |
| BnaA05-107-F | GCGCAGTGGAGAAGATAT | 226 | 12191193 |
| BnaA05-107-R | GAAGGGAGGATGGATGAT |  |  |
| BnaA05-108-F | GGGACCTGGAGTTTGGTT | 234 | 12367465 |
| BnaA05-108-R | AGATCATTGCCTGCGAGA |  |  |
| BnaA05-109-F | GGGATGTGAGTAGCGAATA | 240 | 12153951 |
| BnaA05-109-R | ATACAGGTCAAGCTGGTTT |  |  |
| BnaA05-110-F | TCCTCCAGAAACTCCAAGA | 244 | 12186075 |
| BnaA05-110-R | GCTGCCAAAATGAATAGAA |  |  |
| BnaA05-111-F | AAACTGAAAATGGCAACG | 247 | 12762289 |
| BnaA05-111-R | TTCCAACTCACGGACAAG |  |  |
| BnaA05-112-F | TTCACCGCTCTGATTTCTG | 251 | 12727588 |
| BnaA05-112-R | TCCAAACCTACGGACGAG |  |  |
| BnaA05-114-F | ATGGATAACCCTCGACAAA | 306 | 12570273 |
| BnaA05-114-R | TGGTGGAACTCGCAGAAG |  |  |
| BnaA05-115-F | TTCAAGAGGGGAGATATGAT | 171 | 10321667 |
| BnaA05-115-R | TTGTTTTGGTTTTGTTTCGT |  |  |
| BnaA05-116-F | AACAACATCAAAGCAAACA | 175 | 10494358 |
| BnaA05-116-R | CCTCTACTCATTCCCCAT |  |  |
| BnaA05-118-F | AAGGCTCAAGCATACAGAC | 184 | 10166175 |
| BnaA05-118-R | TATTTTATCCCGTTTTCGT |  |  |
| BnaA05-119-F | GAGTAGAGCTGACGGAGAC | 187 | 11424409 |
| BnaA05-119-R | CCTAACTTTAGCCAAAACA |  |  |
| BnaA05-120-F | CGAGTGCTTTAGTGCCTTAT | 192 | 10399946 |
| BnaA05-120-R | AGATCCAACATCGCCAGA |  |  |
| BnaA05-121-F | ATGCGAAGAAGGTAACTAAA | 200 | 9004691 |
| BnaA05-121-R | GTGGCAAGAGGAGAATAGAA |  |  |
| BnaA05-122-F | AGGAGGTGGGAAAGGATG | 208 | 9083952 |
| BnaA05-122-R | AAAGAACGCCGAAGGAAT |  |  |
| BnaA05-124-F | ACGGAAAAGACATACCTGA | 212 | 11277178 |
| BnaA05-124-R | TTCTAATTGACCGAAAAGC |  |  |
| BnaA05-125-F | TCAGTAGCCAGCCCAGAA | 212 | 11139933 |
| BnaA05-125-R | AGCAAGGATCGGAGAAGG |  |  |
| BnaA05-126-F | CCCAAGATAGGTCCAAAGA | 216 | 9336154 |
| BnaA05-126-R | ATGGAAGAGGTTCGGTTGT |  |  |
| BnaA05-127-F | TGGCAGTTAAGAGGGTTTC | 216 | 9732102 |
| BnaA05-127-R | CTTGTTTAGCCCGAGTGA |  |  |
| BnaA05-128-F | GAGATGTGAGCGACGAAG | 232 | 10878023 |
| BnaA05-128-R | AGAATGTGACTGAGAAACCC |  |  |
| BnaA05-129-F | GTATAATCGGCGGGAAGT | 233 | 11065124 |
| BnaA05-129-R | GTGATGCCAATCTCGTCC |  |  |
| BnaA05-130-F | CCCTATAATCAATCCGTGTA | 253 | 10638084 |
| BnaA05-130-R | CGTAGTGGTTTCAGCAAAG |  |  |
| BnaA05-131-F | GATTGAGGCATGATAGAAG | 274 | 9866772 |
| BnaA05-131-R | TATAATGGGAGGGAAGAAC |  |  |
| BnaA05-132-F | GGAGCAGGCTGATGGAGT | 274 | 9084363 |
| BnaA05-132-R | CAAGCGAAATGGGACAAA |  |  |
| BnaA05-133-F | TCTTCCTTCTTCCTCGTG | 274 | 10139971 |
| BnaA05-133-R | AGCTATTGCTTTCCCTGA |  |  |
| BnaA05-135-F | AATGCTTAACCTTGCTCTATT | 283 | 9074497 |
| BnaA05-135-R | TGGACACCCTAAACACTCAG |  |  |
| BnaA05-136-F | GAAGAATGGGTTGGTGATA | 286 | 9959246 |
| BnaA05-136-R | AAGTACAAGTTTGCGACTG |  |  |
| BnaA05-137-F | CTGAAGAAGAACGGCTAAA | 299 | 10951682 |
| BnaA05-137-R | TGGCATAGGCTGATTGTAG |  |  |
| BnaA05-138-F | GCCATAGTCAAAACCAGAA | 304 | 10706497 |
| BnaA05-138-R | TTGTTAGAAGACTCGCTCC |  |  |
| BnaA05-139-F | AAGCCGACCGTCCGATTA | 170 | 11853953 |
| BnaA05-139-R | GATGCCCGAACTTCTCCC |  |  |
| BnaA05-140-F | ACGGGTTAGCCCTACTTTA | 170 | 11866285 |
| BnaA05-140-R | GGTGCGGATTTCATATTTT |  |  |
| BnaA05-141-F | ACTGCTGTCGCCAATCTTAG | 171 | 11907477 |
| BnaA05-141-R | ATGATGGTTGCGTACCTTTT |  |  |
| BnaA05-142-F | ATAACTCATCCAAGACAGGCT | 174 | 11775671 |
| BnaA05-142-R | AGAAGGCTTAGTTATTTTGGC |  |  |
| BnaA05-143-F | TCTCCCACTGAACCTTACA | 174 | 11777369 |
| BnaA05-143-R | GATTTGCCTTTGGTCTTTT |  |  |
| BnaA05-144-F | CCAAACCAGCAGACACCT | 176 | 11895556 |
| BnaA05-144-R | ATACGCCACAGAGGACGA |  |  |
| BnaA05-145-F | TTAGTGAATACGAATCGGTGAA | 178 | 11521214 |
| BnaA05-145-R | ATGTGCGACGCTGGAGAC |  |  |
| BnaA05-146-F | CGAAATTAAACTAAACCCAA | 181 | 11398704 |
| BnaA05-146-R | GAGACGAAGATTCAACAACA |  |  |
| BnaA05-147-F | GAGTAGAGCTGACGGAGAC | 186 | 11424484 |
| BnaA05-147-R | CTAACTTTAGCCAAAACATC |  |  |
| BnaA05-148-F | TGCTGCTTGCGACTTGAA | 201 | 11822671 |
| BnaA05-148-R | ACCGAGAACCTGCCCATT |  |  |
| BnaA05-150-F | GCATAGCAAGACTGTCCAAC | 215 | 11619028 |
| BnaA05-150-R | AGAAGCAATCAGCAAGAAGA |  |  |
| BnaA05-151-F | TTCAAAATATGCCCGTCA | 225 | 11291932 |
| BnaA05-151-R | AAAGTAACAAACAGGTAGCCA |  |  |
| BnaA05-152-F | ACAGGACGATGCTTCACTC | 231 | 11328271 |
| BnaA05-152-R | AGATTTGCTCGGACTTGC |  |  |
| BnaA05-153-F | ACTGAACTACCATAAAACCAT | 231 | 11508382 |
| BnaA05-153-R | TTGAGTAATCGGAGATCTGA |  |  |
| BnaA05-154-F | CAAACCACAAAATCATACA | 231 | 11839817 |
| BnaA05-154-R | TTTAACCAAACCCACTAAT |  |  |
| BnaA05-155-F | GTGTTAAGAAGAAGTAGACCGAG | 241 | 11850891 |
| BnaA05-155-R | AAAATATGCCCCTTGAAAT |  |  |
| BnaA05-156-F | AAAGGGTAAAGGATGAATT | 245 | 11609618 |
| BnaA05-156-R | ATGAAACGACGCACAGAG |  |  |
| BnaA05-157-F | GGTCGGAGACAGCGTTAC | 247 | 11458041 |
| BnaA05-157-R | AGTGCCTAGTCAGCTTTCAT |  |  |
| BnaA05-158-F | TAAACGCATTGCCAAATA | 249 | 11698869 |
| BnaA05-158-R | GATGGAAAGAACATACCGA |  |  |
| BnaA05-159-F | AGTTTTAGGTGGATGTGCAG | 250 | 11819036 |
| BnaA05-159-R | GTCCCATATTGAGTGACGAT |  |  |
| BnaA05-160-F | CATTTGACCATCATTCCATA | 251 | 11799766 |
| BnaA05-160-R | TTTTCATCGTTCCAAGCA |  |  |
| BnaA05-161-F | GTGCATTCACTGTAAGGGTAA | 258 | 11926688 |
| BnaA05-161-R | CGCTGCCATTCTTCGTAA |  |  |
| BnaA05-162-F | AAACCTCTGCCCAAACCC | 261 | 11844097 |
| BnaA05-162-R | CCAACCGATTCTTCTTCTCC |  |  |
| BnaA05-163-F | CAACATGAGCGGTCAAATA | 266 | 11814695 |
| BnaA05-163-R | GTCTAATGCTTCCAGTGCC |  |  |
| BnaA05-164-F | CCAGGTAAGGAAGAAGGTG | 279 | 11300793 |
| BnaA05-164-R | GGTTGAAGATTGAAGGAGC |  |  |
| BnaA05-165-F | AATGCAAACATATTCAACG | 283 | 11698831 |
| BnaA05-165-R | GATGGAAAGAACATACCGA |  |  |
| BnaA05-166-F | GTGACAATTATGAATCCATCG | 287 | 11834289 |
| BnaA05-166-R | AAAAGACAAATATCCCCAAAA |  |  |
| BnaA05-167-F | CTCGCTTATTGAGTTTCAC | 311 | 11800295 |
| BnaA05-167-R | TAGATGCTGCCATGTTAGT |  |  |
| BnaA05-210-F | GTCACAACCCGCAAACC | 197 | 12147698 |
| BnaA05-210-R | CCTCGCAGTGCTTCCAT |  |  |
| BnaA05-211-F | GTCTTGTCAACAGTGAGGGAG | 175 | 12171245 |
| BnaA05-211-R | AAGCGGCGGAGGATG |  |  |
| BnaA05-212-F | ATCATCCATCCTCCCTTCC | 269 | 12191447 |
| BnaA05-212-R | CTTCGTCGTCGCCTTCTT |  |  |
| BnaA05-213-F | AGGATTTCTTCCCTTTTA | 181 | 12220080 |
| BnaA05-213-R | TGATTTCGGTGTATTTGA |  |  |
| BnaA05-214-F | ATTACGATGAGGGTAGTTT | 216 | 12290232 |
| BnaA05-214-R | GTTAGGACCTTGTTTGATT |  |  |
| BnaA05-215-F | CCAAGCGTGACCTTATCT | 171 | 12338876 |
| BnaA05-215-R | CTTGTGACCACTGAGCCT |  |  |
| BnaA05-216-F | GTCAAGAAAAGAATCCCATA | 297 | 12353142 |
| BnaA05-216-R | CTTAGATCAATAAACGGCTC |  |  |
| BnaA05-217-F | CACAAACCTATCTGAACCCTG | 247 | 12464562 |
| BnaA05-217-R | CCCAAGTCCAAGCCAAA |  |  |
| BnaA05-218-F | AGAAGTCGTTGGGTTAGTA | 255 | 12489518 |
| BnaA05-218-R | AAAAGTGGAAACAGAAGAA |  |  |
| BnaA05-219-F | AACCAGCAAGAAGAAGAG | 229 | 12504165 |
| BnaA05-219-R | ATTTACTGATGCGAAGAC |  |  |
| BnaA05-220-F | TTTTCTGGGACCCTTTCA | 173 | 12507106 |
| BnaA05-220-R | AACCCGTTGGTGCCTAAT |  |  |
| BnaA05-221-F | GTTACGCACCAACCAAATA | 219 | 12536833 |
| BnaA05-221-R | TGAAACAAGGGTTATGGAC |  |  |
| BnaA05-222-F | ACCACATTTTCCCTAACTC | 185 | 12547730 |
| BnaA05-222-R | TCTGCCACCTTGTCTCAC |  |  |
| BnaA05-223-F | ACCCTACCAAGGCAGTCC | 218 | 12576545 |
| BnaA05-223-R | TTGCGGCGAAAGACATAG |  |  |
| BnaA05-224-F | CTTCTCCTTGCTTTGTTTCA | 195 | 12612506 |
| BnaA05-224-R | CTCTGTTCTCGTTTCTTTGC |  |  |
| BnaA05-225-F | TGCCTGACATACCCCTCTAC | 233 | 12634826 |
| BnaA05-225-R | CATTACCGATGACCCCACTA |  |  |
| BnaA05-226-F | TCGGTTAGATGGTGAGGC | 281 | 12657284 |
| BnaA05-226-R | ACGATTTTGGCAGAGGTT |  |  |
| BnaA05-227-F | TGGGTTCTCGTCACTAC | 229 | 12685831 |
| BnaA05-227-R | TGGTTGATTTCCTGCTA |  |  |
| BnaA05-228-F | CACCCTCACATCTTGCTCC | 180 | 12715345 |
| BnaA05-228-R | CTTGGTGACCCGTTCCTC |  |  |
| BnaA05-229-F | GTAATGATGTTCCCTTTTGC | 261 | 12800340 |
| BnaA05-229-R | CTTTCTGGAACTCGTTTAGG |  |  |
| BnaA05-230-F | TTGCCTAAGACGTTAGAAAC | 227 | 12828756 |
| BnaA05-230-R | CATTGAATGGACAATATCATT |  |  |
| BnaA05-231-F | AACTCGGGTCTGAGGGAC | 219 | 12849009 |
| BnaA05-231-R | TTCGGAAACTCGATAGGC |  |  |
| BnaA05-233-F | GTGCAGATGCGACGAAAA | 233 | 12985487 |
| BnaA05-233-R | GGTGAACCGGATGGTGAT |  |  |
| BnaA05-234-F | CCGAACATTGACAGGATT | 170 | 13757679 |
| BnaA05-234-R | CCAAGAACGGTGAAGAGTA |  |  |
| BnaA05-235-F | AGCATTAAGCCACTCCTCA | 173 | 13634245 |
| BnaA05-235-R | CGATAAGCCATCTGTCCAT |  |  |
| BnaA05-236-F | CACGACCACGGTTTCCAC | 181 | 13597963 |
| BnaA05-236-R | CATGTCAAGCCTAATGTCACTC |  |  |
| BnaA05-237-F | TAGGTCCAGAAGTCACCACAT | 180 | 13863866 |
| BnaA05-237-R | GAAACAAAGGGAGTTCAAGATA |  |  |
| BnaA05-238-F | GGAAATGAAAATGGAACAATA | 181 | 13529006 |
| BnaA05-238-R | ACTATTCGTTCCCTGCTTTAT |  |  |
| BnaA05-239-F | GTTTGATTCGGTTCTTCG | 183 | 13489540 |
| BnaA05-239-R | AACGGTTGAGCTTAGTAGGT |  |  |
| BnaA05-241-F | TAGCGGTTACAGCGGTTGA | 187 | 13640787 |
| BnaA05-241-R | ATCCCACAGCTTCTAGTTCGTT |  |  |
| BnaA05-242-F | AAAGCGATAGCCTAATGG | 193 | 13583599 |
| BnaA05-242-R | AAATAGCAGGTTTGTGAGC |  |  |
| BnaA05-243-F | ATGTGATGGTCCGTGGCT | 204 | 13869148 |
| BnaA05-243-R | AGCGGTTTGGTTATGTTCG |  |  |
| BnaA05-244-F | TGACTCCCTTCAACTTTATT | 200 | 13499539 |
| BnaA05-244-R | AGTGACACGCATCTTTCTT |  |  |
| BnaA05-245-F | AGTGGCTGTAAATCTCGTT | 200 | 13874043 |
| BnaA05-245-R | GTTTGGGTTCATCCTTGTG |  |  |
| BnaA05-246-F | CTTAGCCTTAGTGTTTCAGTTT | 201 | 13947395 |
| BnaA05-246-R | GATCTACGGTCGCATTTT |  |  |
| BnaA05-247-F | GACCGTATCTTAGAGTTTCC | 203 | 13692646 |
| BnaA05-247-R | CTCAATCTTTGCTCGTTT |  |  |
| BnaA05-248-F | CCCCTATTCTAGCGAAACGA | 203 | 13821592 |
| BnaA05-248-R | TCAATCTGACTCCCACCAAA |  |  |
| BnaA05-249-F | ACGAGCAAATCCTCCACT | 212 | 13655243 |
| BnaA05-249-R | CTTCTTCCGTCCATTATCTT |  |  |
| BnaA05-250-F | CCTCAGTATCCCGATGTTT | 220 | 13571405 |
| BnaA05-250-R | TGTCCTCCCGTGTATGTTC |  |  |
| BnaA05-251-F | GGTATTGGGCTCAAAGCG | 188 | 13717765 |
| BnaA05-251-R | GAGATGGAGTAACCGTGGC |  |  |
| BnaA05-252-F | TGGAGTAGAGTAGTAGCCAAAG | 240 | 13572070 |
| BnaA05-252-R | GCCACCTAACCACTGATG |  |  |
| BnaA05-253-F | GCCAATGTCTACGAATGA | 250 | 13922672 |
| BnaA05-253-R | GAACCAATAAAGAAAAGGAA |  |  |
| BnaA05-254-F | TGCTCTGCCTCCCGTAAC | 265 | 13547129 |
| BnaA05-254-R | TTGCTCGGACTTGCCATC |  |  |
| BnaA05-255-F | CCATCAGATTTGGCGTTTA | 277 | 13779981 |
| BnaA05-255-R | GTGGTCAGGCATCATTCG |  |  |
| BnaA05-256-F | ATTTGCGAGCCTGATGTA | 286 | 13976183 |
| BnaA05-256-R | AGAGTGGGTAGGAGGGTTA |  |  |
| BnaA05-455-F | TGGAAATGCACTAACCTTG | 168 | 11416418 |
| BnaA05-455-R | GGAGGAAGAAACAGAAGAAC |  |  |
| BnaA05-456-F | AACGGTAACCATGAGTCCT | 168 | 11417047 |
| BnaA05-456-R | AGCAAGATATTTCCCAGAAG |  |  |
| BnaA05-457-F | GATGGTTTATTGGTGGCG | 178 | 11417908 |
| BnaA05-457-R | AATGGAAGAGCTGAGGGAC |  |  |
| BnaA05-458-F | AGATTACTACGAATGGGAGA | 179 | 11421373 |
| BnaA05-458-R | TTGGACACCGATCAAGAG |  |  |
| BnaA05-459-F | TTCCGTTTATACGTTTCC | 185 | 11420625 |
| BnaA05-459-R | ATTTAATGGTTCGTGTCG |  |  |
| BnaA05-460-F | CAGATCGGAAGGGCAAAG | 186 | 11362108 |
| BnaA05-460-R | CCTCACAAACCACCAGCA |  |  |
| BnaA05-461-F | CGTCCGTCAGATGAACAG | 187 | 11357784 |
| BnaA05-461-R | CAGAGCCAAGATAAACAATG |  |  |
| BnaA05-462-F | TGGATAGAATGGAAGCAATAG | 187 | 11350281 |
| BnaA05-462-R | ATGGTAGATGTTTCAAAGCCT |  |  |
| BnaA05-463-F | GTCCCTCAGCTCTTCCAT | 191 | 11417973 |
| BnaA05-463-R | ACGTAAACGTCAGGCTACTT |  |  |
| BnaA05-464-F | TTTAGCACGGGAGCAATC | 191 | 11421462 |
| BnaA05-464-R | CAGAGGAGGCAGGTAGGA |  |  |
| BnaA05-465-F | TTTCATGGTTACTCATATGCTA | 198 | 11353065 |
| BnaA05-465-R | AATAGTGGTAAATGATAACGCT |  |  |
| BnaA05-466-F | GGCTTGTAATACTTTCTTTTC | 200 | 11422176 |
| BnaA05-466-R | CACTTCAGATTGTCACCCT |  |  |
| BnaA05-467-F | AGTTGAACCGCTTGTCCT | 200 | 11359815 |
| BnaA05-467-R | TTGCAATTTTGCATCGTAC |  |  |
| BnaA05-468-F | TCGAAACTCACGACTGGATA | 203 | 11359320 |
| BnaA05-468-R | GGGTGGGATAAAGGAAATAA |  |  |
| BnaA05-469-F | CTCAGGCCAAAGACTCAAA | 204 | 11421019 |
| BnaA05-469-R | GACCTTCCATTCTCAACCC |  |  |
| BnaA05-470-F | CAGCACCAGGAGCTTTATG | 206 | 11418506 |
| BnaA05-470-R | GAAGGAAGTAGCAGGAGTTGT |  |  |
| BnaA05-471-F | AAGTTAGGCGTTGGATTAC | 209 | 11353895 |
| BnaA05-471-R | AAGTTAGGCGTTGGATTAC |  |  |
| BnaA05-472-F | CCCTAAGTAACCGATTGTG | 212 | 11419380 |
| BnaA05-472-R | CATAGTGATAACCTATTCCAGAG |  |  |
| BnaA05-474-F | TATCACCAAATTCTCATCAAC | 220 | 11356966 |
| BnaA05-474-R | TATCACCAAATTCTCATCAAC |  |  |
| BnaA05-475-F | AAAGAGTGAAGAGTTTGGAG | 225 | 11350746 |
| BnaA05-475-R | ATTATCAAAATCATCCTCCT |  |  |
| BnaA05-476-F | GCTGAGCGTCTTCTTACAC | 232 | 11421840 |
| BnaA05-476-R | GCTTACGGAGGAGTTGATC |  |  |
| BnaA05-477-F | TTTGACCTTCTACCGTCTG | 237 | 11349467 |
| BnaA05-477-R | TTAAGTTCGATGGTTCTCG |  |  |
| BnaA05-479-F | GCGTTGTAGTTGTTGTTGG | 248 | 11419739 |
| BnaA05-479-R | CATATTGGAGATGGGGTCA |  |  |
| BnaA05-480-F | CATATGAATCCATCTATCTCG | 250 | 11347215 |
| BnaA05-480-R | CTAAGTCACAAATACCCAAAC |  |  |
| BnaA05-481-F | TGACTATTTCATCGCCCTAT | 252 | 11361123 |
| BnaA05-481-R | TGACTATTTCATCGCCCTAT |  |  |
| BnaA05-482-F | CATTCCTGATTTCGTCTGA | 261 | 11416348 |
| BnaA05-482-R | CTGGTGTAGCAAGTCCTGT |  |  |
| BnaA05-483-F | ACATTGTGGTATTAAAAGGAG | 264 | 11354168 |
| BnaA05-483-R | TAAGGAAGTTGAGTATTGACG |  |  |
| BnaA05-484-F | TGTATTTCCTATCGGTTTCGG | 264 | 11348026 |
| BnaA05-484-R | CTAGATTTGACATGCGCTTCA |  |  |
| BnaA05-485-F | GAGCTAATTTTCAAAGCAAC | 271 | 11416950 |
| BnaA05-485-R | CCTACGCCTGGAAGTCTA |  |  |
| BnaA05-486-F | TCCTCCGCAAAGAAACGC | 274 | 11351593 |
| BnaA05-486-R | GTCCAACGCAGGCCAATC |  |  |
| BnaA05-487-F | AAGTCGTCTAGTAAGTCTTCTGA | 291 | 11355745 |
| BnaA05-487-R | AAATCTACCTTTAAATGAGTGG |  |  |
| BnaA05-488-F | AACCGATTGTGAATACCG | 301 | 11419427 |
| BnaA05-488-R | ACGCATGTATCCGAGTGT |  |  |
| BnaA05-489-F | TTGATTCAACTCTGCCTTAA | 304 | 11418977 |
| BnaA05-489-R | AACTACCAAATACACCAGCA |  |  |
| BnaA05-490-F | AAACAGTGGTGATTAACGTC | 305 | 11353370 |
| BnaA05-490-R | ATCCTTGCTATTAGATGAAAC |  |  |
| BnaA05-321-F | GACATTATTGTTTTGCTTTCAT | 187 | 11078833 |
| BnaA05-321-R | ATCCTCCTCATCATCACATCTA |  |  |
| BnaA05-322-F | TATTGAGCCCAAGAAGAAA | 190 | 13601293 |
| BnaA05-322-R | AACGCTAGGGATATTACATT |  |  |
| BnaA05-324-F | CATCATTTGCTTTTCTTCC | 195 | 11127950 |
| BnaA05-324-R | CGATAGGGTTTGGTTGTC |  |  |
| BnaA05-328-F | TCTGAAGTCTTTGCGTCTC | 218 | 11168723 |
| BnaA05-328-R | AAGTAATGGTTCCGTGTCT |  |  |
| BnaA05-330-F | AATGTCAAACAGACTAATCCTC | 222 | 11037471 |
| BnaA05-330-R | TTCTCGAAACCGACTCAG |  |  |
| BnaA05-331-F | TTCCCTCAGTATCCCGATGT | 228 | 13571415 |
| BnaA05-331-R | TGTCCTGTCCTCCCGTGT |  |  |
| BnaA05-333-F | TGGTAGATTTGGTTCTTTCG | 240 | 11199795 |
| BnaA05-333-R | TTTTGGCAGTGTTATTAGGC |  |  |
| BnaA05-335-F | GCTATCTCCCGTCTTCTC | 246 | 10955733 |
| BnaA05-335-R | GCGGATTATTACCTAGTTATT |  |  |
| BnaA05-336-F | ACATTCTTTCGCTCTGTATCTC | 204 | 11341200 |
| BnaA05-336-R | GTCGATGATCAGTCACCACTAG |  |  |
| BnaA05-337-F | AAAGGAAGATTAGTTTTGTGTT | 283 | 13582725 |
| BnaA05-337-R | AAGCACATTTTGCCTCTAA |  |  |
| BnaA05-340-F | AACTAAAGTGTCTGATTCCAA | 160 | 11638878 |
| BnaA05-340-R | TTCATAGAACAAATCAAAACAT |  |  |
| BnaA05-341-F | CAACCAAACATTCACAACA | 179 | 11584911 |
| BnaA05-341-R | GTCCTCTACGAAGGAGATAA |  |  |
| BnaA05-343-F | CCTCGCTATCTCCCTCTT | 188 | 11721178 |
| BnaA05-343-R | GGTAGTTATCCTTATGTTATTAGTT |  |  |
| BnaA05-344-F | CATCTGACGATAGAACACCCAT | 194 | 13739358 |
| BnaA05-344-R | AACGACGAAAACCCTCCC |  |  |
| BnaA05-347-F | AAAACCCAGAAAGCATATC | 205 | 11712705 |
| BnaA05-347-R | TAGCTCACCTTAAAATCCC |  |  |
| BnaA05-350-F | GCATAGCAAGACTGTCCAAC | 218 | 11619002 |
| BnaA05-350-R | AGAAGCAATCAGCAAGAAGA |  |  |
| BnaA05-351-F | GTAGGGAAATAGCAAACGATA | 223 | 11697041 |
| BnaA05-351-R | CAGGGAGAATGTTTATGAAGT |  |  |
| BnaA05-355-F | TGAGCCATGATGTTTTGTT | 248 | 11543153 |
| BnaA05-355-R | GTTTGATGTCCTCGTTGAT |  |  |
| BnaA05-357-F | TAGCATCTGAGGCTTGTATT | 268 | 11626939 |
| BnaA05-357-R | ATAGAACTTTGGTTTTGATTG |  |  |
| BnaA05-358-F | ACTTCGGCTTCACTCATCCT | 273 | 11559031 |
| BnaA05-358-R | GAATTAACATTTCGGCATCG |  |  |
| BnaA05-364-F | AAGATCCACCCGACTGACC | 183 | 11424594 |
| BnaA05-364-R | CGGAATCCTCTGGCTGAC |  |  |
| BnaA05-368-F | GCAAGAAGTGACTAAGTCCATA | 204 | 11557116 |
| BnaA05-368-R | TTCGTCCAACTCATAACAGAT |  |  |
| BnaA05-370-F | GAAGCAACGCCTTGTAAC | 212 | 11457075 |
| BnaA05-370-R | CAAATCCAAATGGGAGAAA |  |  |
| BnaA05-371-F | TCACTAAACTAAATCTCAGGAA | 212 | 11483438 |
| BnaA05-371-R | TCACTAAACTAAATCTCAGGAA |  |  |
| BnaA05-372-F | TATGTGGAGGAATCTATTGG | 214 | 11530811 |
| BnaA05-372-R | GAAGAGCCTGTTCTTATGC |  |  |
| BnaA05-374-F | TTGGTAATGGCTTCTTCTAC | 222 | 11533645 |
| BnaA05-374-R | AATTTTGGATCTCATGTCTCT |  |  |
| BnaA05-375-F | CGCCATACACTTCACCAG | 224 | 11557708 |
| BnaA05-375-R | ACCTCCAACCCTCCTTCA |  |  |
| BnaA05-377-F | AGCGTTACTACAGACGGACAT | 232 | 11458050 |
| BnaA05-377-R | ACAGTGCCTAGTCAGCTTTCA |  |  |
| BnaA05-379-F | TGAGCCATGATGTTTTGTT | 248 | 11543153 |
| BnaA05-379-R | GTTTGATGTCCTCGTTGAT |  |  |
| BnaA05-380-F | ACACCTACTACATCCAGTGTTG | 173 | 11070074 |
| BnaA05-380-R | CTTGTCCACCTGAGTTAGAATA |  |  |
| BnaA05-381-F | CGAGAAGTATGTCAAGTCGT | 173 | 11117839 |
| BnaA05-381-R | GAAGCATTGTTTCAGATTATT |  |  |
| BnaA05-384-F | TCCGATGTTTTGATGAATA | 189 | 11169056 |
| BnaA05-384-R | TTTGTTTTGGTTACCTGATAC |  |  |
| BnaA05-385-F | ATCCCGCAAATACAAGAG | 191 | 11240276 |
| BnaA05-385-R | AACGAGCAGATCAATACCA |  |  |
| BnaA05-387-F | CCCAGATGGTGGCGTAAA | 198 | 11063344 |
| BnaA05-387-R | TCAGTATGATTCCTTGTTGTTCA |  |  |
| BnaA05-389-F | CCGAGCAGCCAGCAAATA | 204 | 11205852 |
| BnaA05-389-R | CGAGCAGATCAATCAATACCAT |  |  |
| BnaA05-390-F | GCCTCCGCATGAAGGTAC | 206 | 11171667 |
| BnaA05-390-R | AATGGCTGTCTCAACAATCTACT |  |  |
| BnaA05-391-F | CATAAAAGGCGACGACAAC | 209 | 11065114 |
| BnaA05-391-R | CTCCTCCTCGATAAGTACACC |  |  |
| BnaA05-398-F | GATCCTCTGCGTATGGTAC | 283 | 11298483 |
| BnaA05-398-R | GATGTGGCTTATGCTTCTAG |  |  |
| BnaA05-399-F | AAGAAGCCCACAATACAAT | 286 | 11401688 |
| BnaA05-399-R | AGAAGACGAAGAAAGGAGG |  |  |
| BnaA05-406-F | TATTACGAGTTATGGTTTGATT | 182 | 11378158 |
| BnaA05-406-R | TATTAAGTGATATTTTAGGGGA |  |  |
| BnaA05-409-F | ACTCGTCATTCTGATGGTCT | 191 | 11377380 |
| BnaA05-409-R | AGCTTATGGCTCTGCTTAA |  |  |
| BnaA05-410-F | TTTCCTTTGAATGTCGTTT | 191 | 11383624 |
| BnaA05-410-R | TGCCCGTTTAGAATGTTAT |  |  |
| BnaA05-413-F | TACCTTGGCTGGTTGTCC | 199 | 11366469 |
| BnaA05-413-R | AGAACTTGCGGCTGTAAAA |  |  |
| BnaA05-414-F | AATTTGTCCACATCTTACTGGT | 205 | 11341582 |
| BnaA05-414-R | CTTGTTTCTGTTATAGCTCCG |  |  |
| BnaA05-418-F | CGGTTCTGGTTATCCGTAT | 224 | 11397256 |
| BnaA05-418-R | TATTGGTATTCTTAAAGTATGGTC |  |  |
| BnaA05-422-F | GATGTTTCGTACCGAACTAC | 240 | 11381729 |
| BnaA05-422-R | GAGATTTTCTTTTCCTTTATGT |  |  |
| BnaA05-423-F | CGGATTCCCAAAGTTTCTC | 246 | 11370612 |
| BnaA05-423-R | ACGGTCCATTATCGCACAT |  |  |
| BnaA05-424-F | ACGGTTAGAACATTGGTATGT | 253 | 11341938 |
| BnaA05-424-R | GTTTCCGAAATTGGTAGAGA |  |  |
| BnaA05-426-F | AAGTAACTTCAAAGTTATGGATT | 260 | 11343080 |
| BnaA05-426-R | TATTCGCACTCTTCAGCA |  |  |
| BnaA05-427-F | TCTCTTATCACCTTTACTTTGA | 261 | 11371534 |
| BnaA05-427-R | TTGTTGCCATCTTTATTTTC |  |  |
| BnaA05-435-F | TTGGAGAAGCAATTAGATAAGA | 187 | 11414528 |
| BnaA05-435-R | CAAGTTACCATTCAAAAGTGTT |  |  |
| BnaA05-438-F | TCCGACATGCCTCATAAA | 192 | 11400798 |
| BnaA05-438-R | CAAGACTCCGTAGACAACAAC |  |  |
| BnaA05-439-F | CCATTTTATTCCATTTTGATTC | 196 | 12472477 |
| BnaA05-439-R | TAATTTGCTTTGGTGGATAACT |  |  |
| BnaA05-440-F | CTCACTAAGGTCAGTTTGATATG | 200 | 11414146 |
| BnaA05-440-R | TTCTCCAAGTTATGAAAGTTATT |  |  |
| BnaA05-442-F | TGTTTCTTCCCCAACCACTC | 206 | 11415503 |
| BnaA05-442-R | TCCCAAATTCTTTATGTTACTGC |  |  |
| BnaA05-443-F | TGTGGGCTTGATCCTTGTTAT | 213 | 11419981 |
| BnaA05-443-R | AGCGGGTGCAGGTAGAAAT |  |  |
| BnaA05-444-F | GCTCGGGACAAAATGAAT | 216 | 11415667 |
| BnaA05-444-R | CAAAACGCCAATAACTGCT |  |  |
| BnaA05-445-F | TGACCAGCTCTTCATCCTC | 233 | 11423551 |
| BnaA05-445-R | ATCCGCAACTTCTCCTTC |  |  |
| BnaA05-448-F | CGTGGAGATTTTCTGTAAC | 253 | 11407537 |
| BnaA05-448-R | CCCTAAGACATCTTTGGTT |  |  |
| BnaA05-450-F | TTATGAAAATTGGTACGCG | 263 | 11407584 |
| BnaA05-450-R | AGGGTTGTTGAACTTGGAA |  |  |
| BnaA05-452-F | GTTTCGTCGGGAGTTAGA | 275 | 11401559 |
| BnaA05-452-R | TTAGTTGTAGTTGCAGGTTGA |  |  |
| BnaA05-453-F | GTCCATAAGAAGACATCCACC | 279 | 11363303 |
| BnaA05-453-R | TTAAATCTAACATTTTGTACCCC |  |  |
| BnaA05-730-F | CTCTACTCGGTCCTTCTCTTCTT | 257 | 11424235 |
| BnaA05-730-R | CTCTTTCTTTGAAGGAGAGGAGT |  |  |
