# Supplementary material for: Fine mapping of the BnUC2 locus related to leaf up-curling and plant semi-dwarfing in Brassica napus
Source: BMC Genomics. 2020 Jul 31;21:530. doi: 10.1186/s12864-020-06947-7 (PMC7430850; doi:10.1186/s12864-020-06947-7)
Supplement: Supplementary file 3 — Additional file 3 : Figure S1. Frequency distribution of plant height in the BC5F3 (1) and BC5F3 (2) populations. [file 12864_2020_6947_MOESM3_ESM.docx]

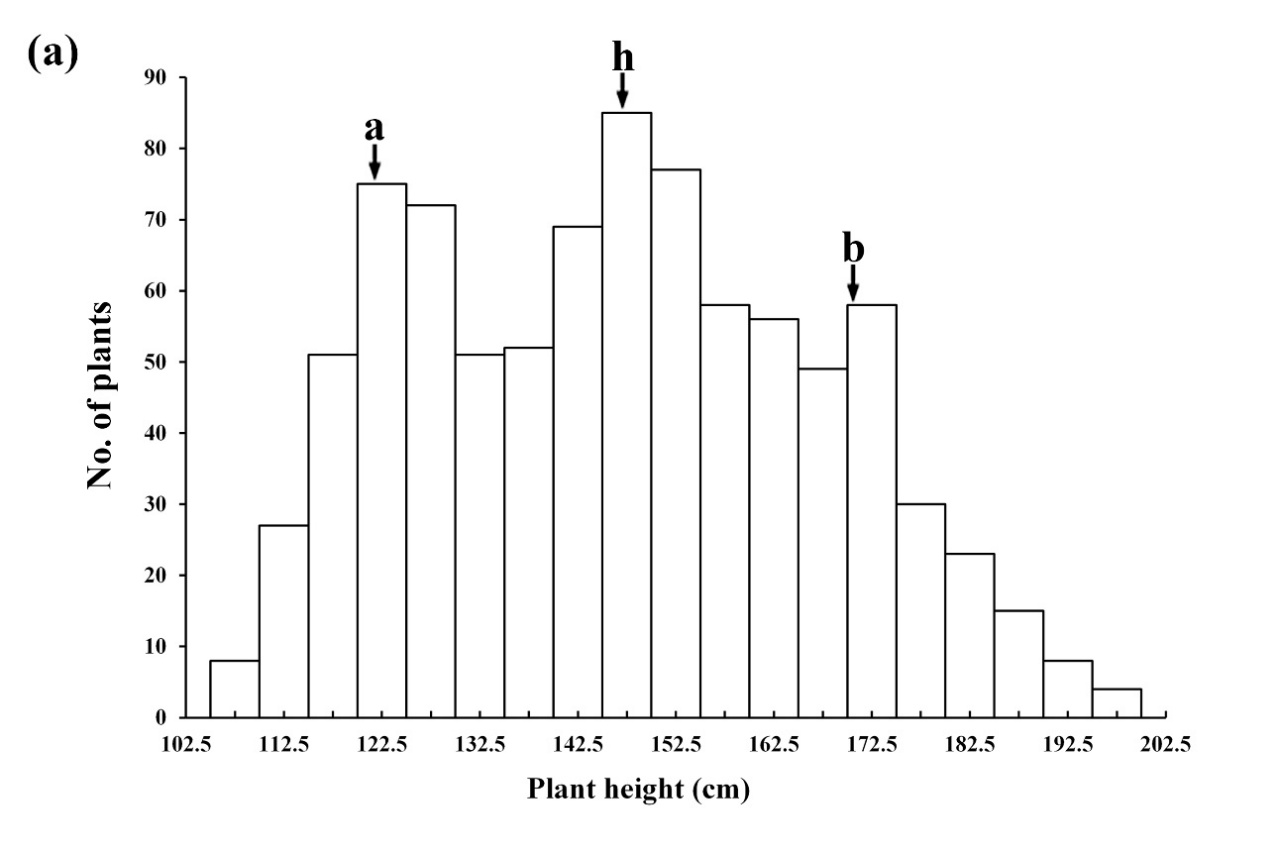

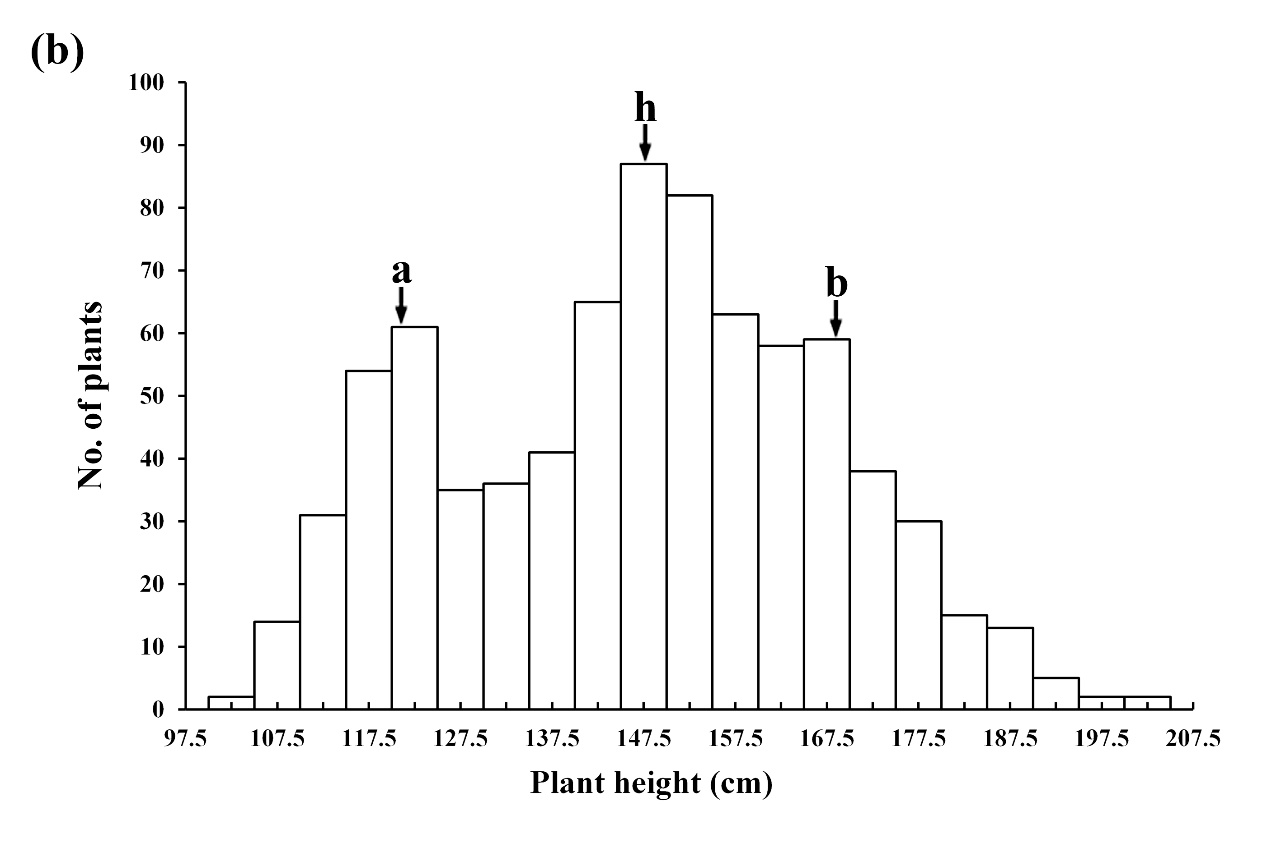


**Additional file 3: Fig S1** Frequency distribution of plant height in the BC_5_F_3_ (1) and BC_5_F_3_ (2) populations. The a indicates the average height of plants with homozygous *BnUC2*, h indicates the average height of plants with heterozygous *BnUC2*, and b indicates the average height of flat leaf plants. The (a) and (b) show the frequency distribution of plant height in the BC_5_F_3_ (1) and BC_5_F_3_ (2) populations, respectively.
