# Supplementary material for: Fine mapping of the BnUC2 locus related to leaf up-curling and plant semi-dwarfing in Brassica napus
Source: BMC Genomics. 2020 Jul 31;21:530. doi: 10.1186/s12864-020-06947-7 (PMC7430850; doi:10.1186/s12864-020-06947-7)
Supplement: Supplementary file 4 — Additional file 4 : Table S3. The designed primers of comparative sequencing and CAPS marker used in this study. [file 12864_2020_6947_MOESM4_ESM.docx]

**Additional file 4: Table S3** The designed primers of comparative sequencing and CAPS marker used in this study.

| Name of Primers | Sequence of primers | Product length |
| --- | --- | --- |
| BnaA05g16680-F | ATGATCGGACAGCTTATTAACCTC | 1929 bp |
| BnaA05g16680-R | TCAGGATCTGTTCTTGCAGTACTTC |  |
| BnaA05g16690-F | ATGTATGGTTTTTTCTCGAACTTTC | 736 bp |
| BnaA05g16690-R | GGTTAATTACATTAGTGTTTTCAGTGCAACC |  |
| BnaA05g16700-F | ATGGCGTACGAGAAAGTCAACG | 850 bp |
| BnaA05g16700-R | AGTGTTCGTCAGGAATATTGC |  |
| BnaA05g16710-F | ATGTTGTTGAATCTTCGTCTCGATGGAAGT | 2556 bp |
| BnaA05g16710-R | CTAAAGCTCATCATCACAATCTTCAGTATTGCTC |  |
| BnaA05g16720-F | ATGGACTCCCTTCCTCAACCGC | 1272 bp |
| BnaA05g16720-R | TTACATGACGTCATTGTGCCATGAGA |  |
| CAPS0-F | CTCCTACGAAGTAAGTAAATATCTGAG | 186 bp |
| CAPS0-R | TCCGTCCATACTCACTTTCACAT |  |
