# Supplementary material for: Fine mapping of the BnUC2 locus related to leaf up-curling and plant semi-dwarfing in Brassica napus
Source: BMC Genomics. 2020 Jul 31;21:530. doi: 10.1186/s12864-020-06947-7 (PMC7430850; doi:10.1186/s12864-020-06947-7)
Supplement: Supplementary file 5 — Additional file 5 : Figure S2. Part of marker experimental results for CAPS marker. [file 12864_2020_6947_MOESM5_ESM.docx]

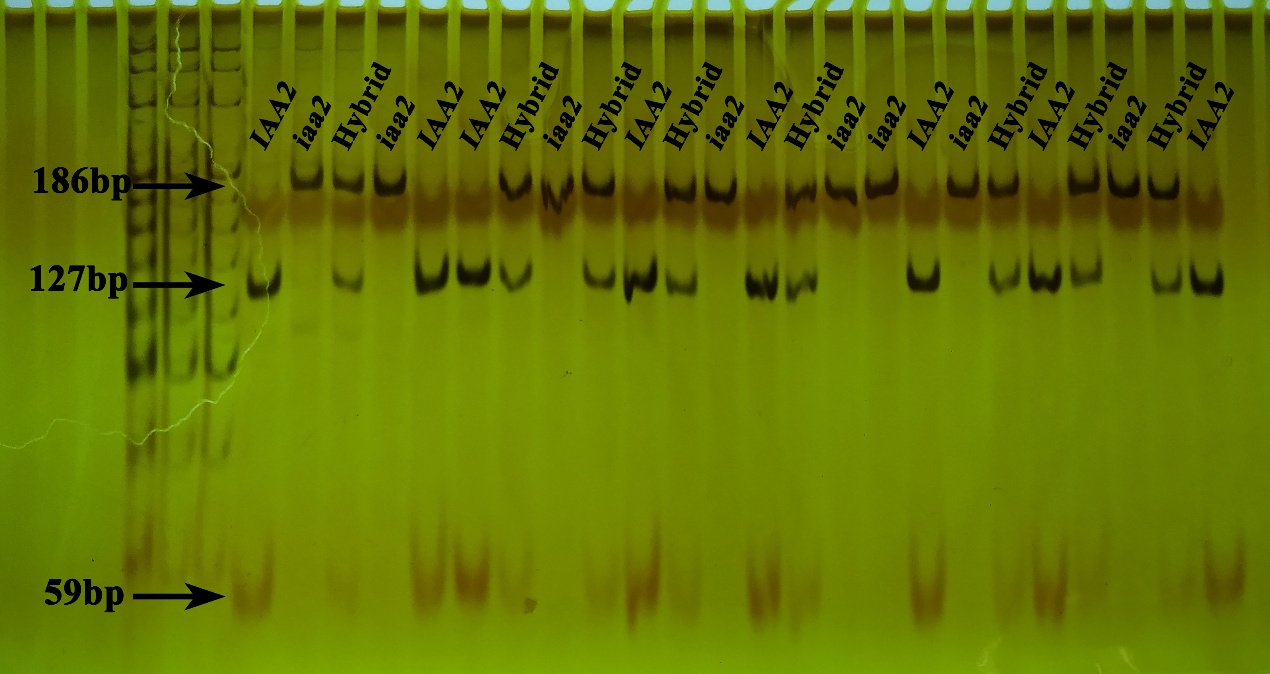


**Additional file 5: Fig S2** Part of marker experimental results for CAPS marker. The variant site on the Degron motif (GWPPV) of *BnaA05.IAA2* were completely co-segregated with the phenotypes in the BC_5_F_3_ population. The polymorphic bands of a CAPS marker were detected among *iaa2* (homozygous plants with up-curled leaves), *IAA2* (plants with flat leaves), and the Hybrid (heterozygous plants with up-curled leaves) by polyacrylamide gel electrophoresis.
