# Supplementary material for: Fine mapping of the BnUC2 locus related to leaf up-curling and plant semi-dwarfing in Brassica napus
Source: BMC Genomics. 2020 Jul 31;21:530. doi: 10.1186/s12864-020-06947-7 (PMC7430850; doi:10.1186/s12864-020-06947-7)
Supplement: Supplementary file 6 — Additional file 6 : Figure S3. Full-length, original blots image of Fig. 3. [file 12864_2020_6947_MOESM6_ESM.docx]

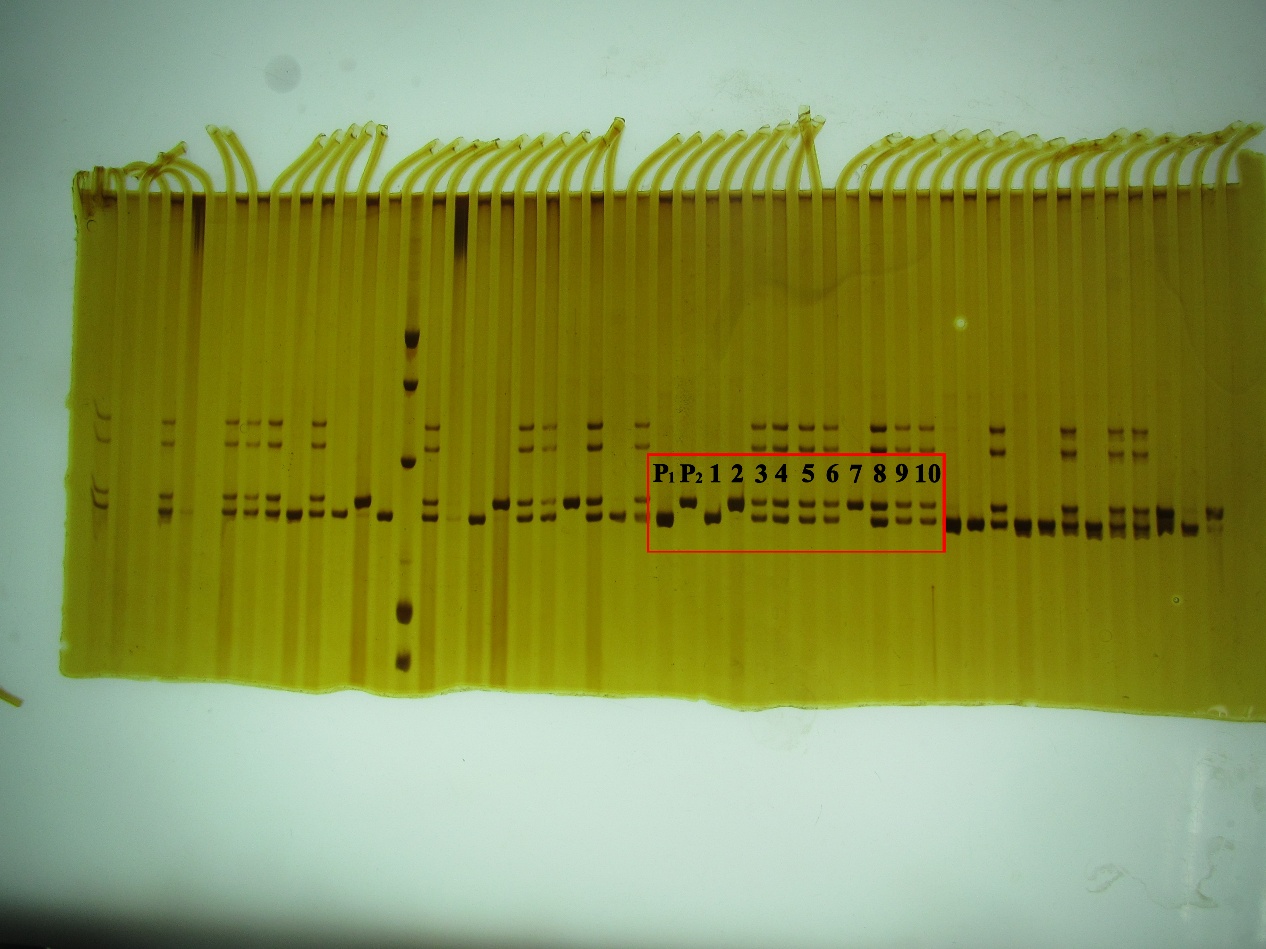


Additional file 6: Fig S3. Full-length, original blots image of Fig. 3. The red box represents the cropped image of Fig. 3. Marker scan with progeny BC_5_F_3_ populations derived from the parents ZS11 and NJAU5737 was conducted. P_1_ and P_2_ indicates PCR products from the parents ZS11 and NJAU5737 plants, respectively. The number 3, 4, 5, 6, 8, 9 and 10 denote the PCR products from heterozygous plants with up-curled leaves, and 1 denote the PCR products from homozygous plants with up-curled leaves, and 2 and 7 denote the PCR products from homozygous plants with flat leaves.
